# Time‐restricted feeding mitigates Alzheimer's disease‐associated cognitive impairments via a *B. pseudolongum*‐propionic acid‐FFAR3 axis

**DOI:** 10.1002/imt2.70006

**Published:** 2025-02-21

**Authors:** Yihang Zhao, Mengzhen Jia, Chen Ding, Bingkun Bao, Hangqi Li, Jiabin Ma, Weixuan Dong, Rui Gao, Xuhui Chen, Jiao Chen, Xiaoshuang Dai, Yuanqiang Zou, Jun Hu, Lin Shi, Xuebo Liu, Zhigang Liu

**Affiliations:** ^1^ College of Food Science and Engineering Northwest A&F University Yangling China; ^2^ The First Affiliated Hospital of Xi'an Jiaotong University Xi'an China; ^3^ Peking University Shenzhen Hospital Shenzhen China; ^4^ Xbiome Shenzhen China; ^5^ BGI Research Shenzhen China; ^6^ Shaanxi Normal University Xi'an China; ^7^ Northwest A&F University Shenzhen Research Institute Shenzhen China

**Keywords:** Alzheimer's disease, gut microbiota, propionic acid, time‐restricted feeding

## Abstract

Time‐restricted feeding (TRF) holds promise for alleviating cognitive decline in aging, albeit the precise mechanism via the gut‐brain axis remains elusive. In a clinical trial, we observed, for the first time, that a 4‐month TRF ameliorated cognitive impairments among Alzheimer's disease (AD) patients. Experiments in 5xFAD mice corroborated the gut microbiota‐dependent effect of TRF on mitigating cognitive dysfunction, amyloid‐beta deposition, and neuroinflammation. Multi‐omics integration linked *Bifidobacterium pseudolongum* (*B. pseudolongum*) and propionic acid (PA) with key genes in AD pathogenesis. Oral supplementation of *B. pseudolongum* or PA mimicked TRF's protective effects. Positron emission tomography imaging confirmed PA's blood‐brain barrier penetration, while knockdown of the free fatty acid receptor 3 (FFAR3) diminished TRF's cognitive benefits. Notably, we observed a positive correlation between fecal PA and improved cognitive function in an AD cohort, further indicating that TRF enhanced PA production. These findings highlight the microbiota‐metabolites‐brain axis as pivotal in TRF's cognitive benefits, proposing *B. pseudolongum* or PA as potential AD therapies.

## Highlights

- Time‐restricted feeding (TRF) improved cognitive function in Alzheimer's disease (AD) patients, with notable enhancement in executive function.
- Multi‐omics integrated analysis in AD mice identified *Bifidobacterium pseudolongum* (*B. pseudolongum*) and propionic acid (PA) as key mediators of TRF's cognitive benefits.
- The potential molecular mechanism by which TRF alleviates cognitive impairment induced by AD involves the *B. pseudolongum*–PA‐free fatty acid receptor 3 (FFAR3) pathway.
- Case‐control study and TRF clinical intervention demonstrated PA as a potential biomarker for AD.

## Graphical abstract

A 4‐month of time‐restricted feeding (TRF) intervention alleviated cognitive impairments in Alzheimer's disease (AD) patients, while a 3‐month TRF regimen improved spatial memory, reduced amyloid‐beta accumulation, and promoted microglial aggregation around plaques in AD mice. Antibiotic‐induced gut microbiota depletion partly abolished TRF's benefits. Through creatively integrating gut microbiota, metabolites, and hippocampal genes, *Bifidobacterium pseudolongum* (*B. pseudolongum*) and propionic acid (PA) were identified as key contributors to TRF's cognitive effects, with supplementation of either mimicking TRF's protective benefits. Positron emission tomography imaging revealed that PA directly crossed the blood‐brain barrier, and PA supplementation restored disrupted metabolism in AD mice. Knockdown of its receptor free fatty acid receptor 3 (FFAR3) diminished TRF's protective effects. A case‐control study showed a negative association between PA and cognitive status, while the TRF clinical intervention linked fecal PA to cognitive status. These findings suggest PA as a potential biomarker and underscore precise TRF‐based nutritional interventions as a promising strategy for managing neurodegenerative diseases.

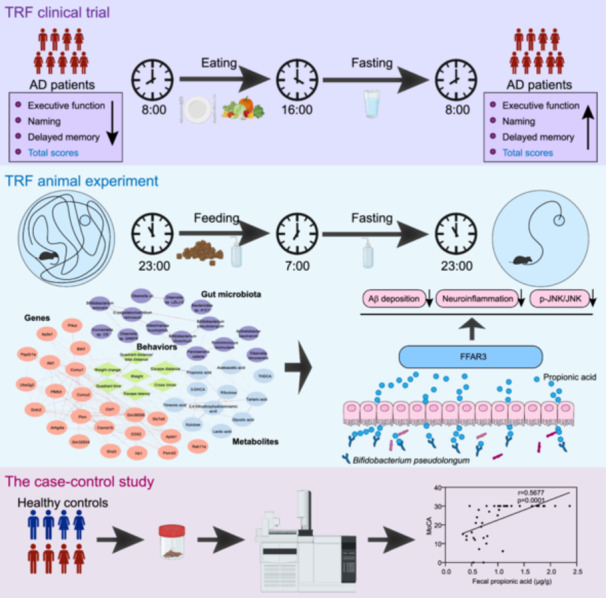

## INTRODUCTION

The Alzheimer's disease (AD) epidemic continues its relentless advance, affecting over 400 million people worldwide. Although AD pathogenesis is still unclear, the deposition of amyloid‐beta (Aβ) protein deposition and tau protein fiber entanglement are classic pathological hallmarks of AD, which subsequently cause cognitive dysfunction, neuroinflammation, and neuronal damage [1, 2]. Accumulating experimental and clinical data have revealed the role of the gut microbiota‐brain axis in AD progression [3, 4]. Improving understanding of the gut‐brain axis holds great potential for developing microbial‐based interventions and therapeutic strategies for the prevention and management of AD and other neuropsychiatric disorders. AD is intricately linked with a dysbiotic alteration of gut microbiota, marked by an elevation in pro‐inflammatory phyla and a reduction in anti‐inflammatory phyla, coupled with modifications in microbial metabolite compositions, including short‐chain fatty acids (SCFAs), branched‐chain amino acids, and bile acids [5]. This intricate interplay between gut microbiota and brain function underscores the potential therapeutic avenues targeting microbial ecosystems in the management and prevention of AD, paving the way for innovative strategies in neurodegenerative disease research.

Time‐restricted feeding (TRF) is a safe and effective fasting regimen that limits food intake to 8‐10 h a day without limiting the amount of food and nutrients consumed. Compared with traditional fasting modes like calorie restriction and 24‐h fasting, TRF is gentler, easier to adhere to, and more suitable for individuals, which has been employed in limited clinical trials for the management of obesity and diabetes [6, 7]. Recent studies highlight the neuroprotective potentials of TRF in neurodegenerative diseases, including subcortical vascular dementia and Huntington's disease, through regulating oxidative stress, glucose and lipid metabolism, autophagy, and microglia differentiation pathway [8, 9, 10, 11]. The latest research also underscores the importance of restoring circadian rhythm dysregulation in TRF to augment cognitive function in AD model mice [12]. While numerous studies have reported the beneficial effects of TRF, most existing evidence is primarily based on mouse models and the precise mechanisms, especially the role of gut microbiota, underlying these benefits remain elusive. Our previous research showed that fasting effectively alleviates diabetes‐induced cognitive impairment by regulating gut microbiota [13]. TRF exerts profound effects on altering gut microbiome and microbial metabolites, predominantly observed in animal models and limited human trials [13, 14, 15, 16]. Although there is consistent evidence on the gut microbiota‐metabolite‐brain axis, our understanding of specific bacteria and their metabolites that contribute to cognitive enhancement remains limited [17]. Additionally, it is crucial to acknowledge that most of these studies have been conducted in mice, thus restricting their direct translation to human applications. There is thus an urgent need for comprehensive investigations to establish the theoretical foundation underlying dietary strategies aimed at improving gut and brain health.

In the current study, we conducted comprehensive investigations to decipher the therapeutic potential of TRF in ameliorating cognitive impairment associated with AD. For the first time, a 4‐month TRF intervention significantly improved the cognitive status of AD patients. A 3‐month TRF successfully delayed the progression of AD pathology in the transgenic mice with five familial Alzheimer's disease (5xFAD) mice, an animal model that rapidly recapitulates only the amyloid pathology of AD. Multi‐omics integrative analyses and the subsequent antibiotic treatment demonstrated the essential role of gut microbiota‐metabolite‐brain axis in mediating TRF‐dependent benefits on cognitive dysfunction in AD mice. Colonization of AD mice with *Bifidobacterium pseudolongum* (*B. pseudolongum*), a probiotic bacterium significantly promoted by TRF, could mimic the ameliorating activity of TRF on AD‐induced cognitive dysfunction via producing propionic acid (PA) with specific targets on free fatty acid receptor 3 (FFAR3) activation. Furthermore, a significant association between cognitive functions and fecal PA level was demonstrated in a cross‐sectional population as well as in the abovementioned intervention trial. Our study unequivocally provides novel and compelling evidence that proves the fundamental linkage of the gut microbiome and microbial metabolites with TRF and AD physiology and also demonstrates the therapeutic potential of *B. pseudolongum* or PA as a nutritional therapy for AD treatment and management.

## RESULTS

### TRF mitigates cognitive dysfunction in AD patients and mice model

**Figure 1:**
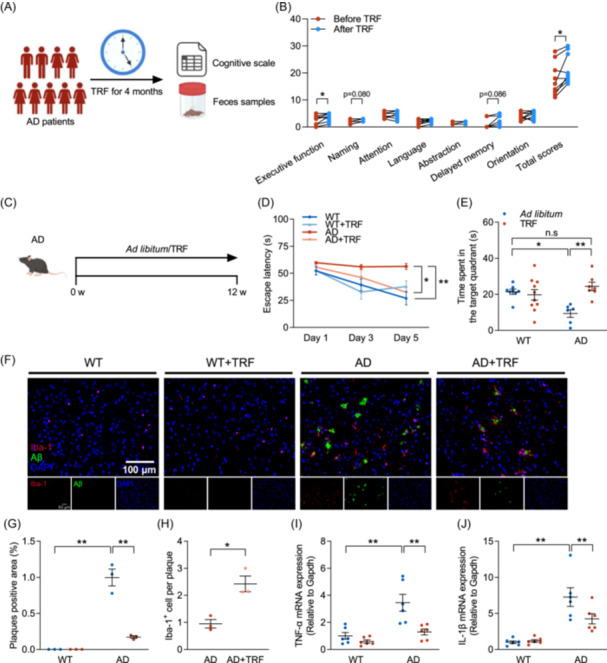
Time‐restricted feeding (TRF) alleviates cognitive impairments in Alzheimer's disease (AD). (A) Schematic of 4‐month TRF intervention in AD patients (*n* = 9). (B) Quantification of montreal cognitive assessment (MoCA) total scores and sub items (executive function, naming, attention, language, abstraction, delayed memory, orientation) from AD patients before and after TRF intervention. (C) Schematic of the treatment with TRF or *ad libitum* in each group (*n* = 6–10). (D) Escape latency. (E) Time spent in the target quadrant. (F) Amyloid‐beta (Aβ) deposition (green) and ionized calcium‐binding adaptor molecule 1 (Iba‐1^+^) (red) microglia immunohistochemical fluorescence images on mice cortex (*n* = 3) (Scale bar, 100 μm). (G) Quantification of plaques positive area. (H) Quantification of Aβ plaque‐associated microglia. (I) mRNA level of tumor necrosis factor‐α (TNF‐α) (*n* = 6). (J) mRNA level of interleukin‐1β (IL‐1β) (*n* = 6). Data are the means ± SEM. *p < 0.05, **p < 0.01; two‐way ANOVA with Tukey multiple comparisons test.

Current evidence on the neuroprotective effects of TRF primarily consists of animal studies, with a scarcity of human data. Here, we first conducted a 4‐month small population TRF clinical intervention trial to assess its impact on cognitive functions in AD patients (*n* = 9) (Figure 1A). Cognitive function tests demonstrated that TRF significantly enhanced the montreal cognitive assessment (MoCA) scores of AD patients, particularly in areas of executive function (*p* < 0.05), underscoring the mitigating impacts of TRF intervention on cognitive impairment in AD patients (Figure 1B).

To investigate the biological mechanism underlying the protective effects of TRF on AD‐caused cognitive impairments and pathological damage, 6‐month‐old 5xFAD mice were fed either *ad libitum* or fasted for 16 h per day for 3 months (Figure 1C). The Morris water maze test showed that escape latency was significantly increased in AD mice during 5‐day initial spatial training compared with WT mice, which was reversed by TRF intervention (Figure 1D). TRF robustly elevated the time spent in the target quadrants (Figure 1E) and the proportion of distance in the target quadrant (Figure S1F) in AD mice to the normal level as in WT mice, proving the restoring effects of TRF on the spatial memory of AD mice. Results from immunofluorescence staining demonstrated considerable aggregation of Aβ and ionized calcium‐binding adaptor molecule 1 (Iba‐1) in the cortex and hippocampus of AD mice, confirming AD‐induced amyloid deposition and microglia activation (Figure 1F and Figure S1H). TRF intervention successfully reduced the positive area of plaques and increased the clustering of microglia in AD mice (Figure 1G,H and Figure S1I,J), accompanied by a reduction in mRNA expressions of tumor necrosis factor‐α (TNF‐α) and interleukin‐1β (IL‐1β) (Figure 1I,J). Moreover, TRF intervention greatly increased the mRNA expression of brain‐derived neurotrophic factor (BDNF) in the cortex of AD mice, a key gene critical for the survival, maintenance, and regeneration of specific neuronal populations in the brain (Figure S1K). Our findings collectively demonstrate that TRF enhances cognitive memory function in both AD patients and AD mice. TRF significantly mitigates the pathological manifestations of AD by promoting the recruitment of microglia around Aβ plaques and concurrently reducing Aβ deposition and neuroinflammation.

### Integrated multi‐omics analysis demonstrates the gut microbiota‐metabolite‐brain axis underlying the TRF‐dependent effects on cognitive dysfunction in AD mice

Prior studies, albeit limited, have demonstrated encouraging outcomes of fasting on neurobiological health, which is in line with our findings [12, 17]. Nevertheless, the mechanisms underlying how TRF ameliorates AD remain elusive. The gut microbiota communicates with the brain through complex bidirectional communication systems—the gut‐brain axis, which integrates peripheral intestinal function and microbial metabolism with emotional and cognitive brain centers via neuro‐immuno‐endocrine mediators [17, 18, 19]. Considering the increasing connection between gut microbiota and microbial metabolites with AD pathogenesis, the improved understanding of the benefits of TRF on AD and how the gut microbiota‐metabolite‐brain axis is involved in this process is critically important for the prevention and management of AD and other neurodegenerative diseases.

**Figure 2:**
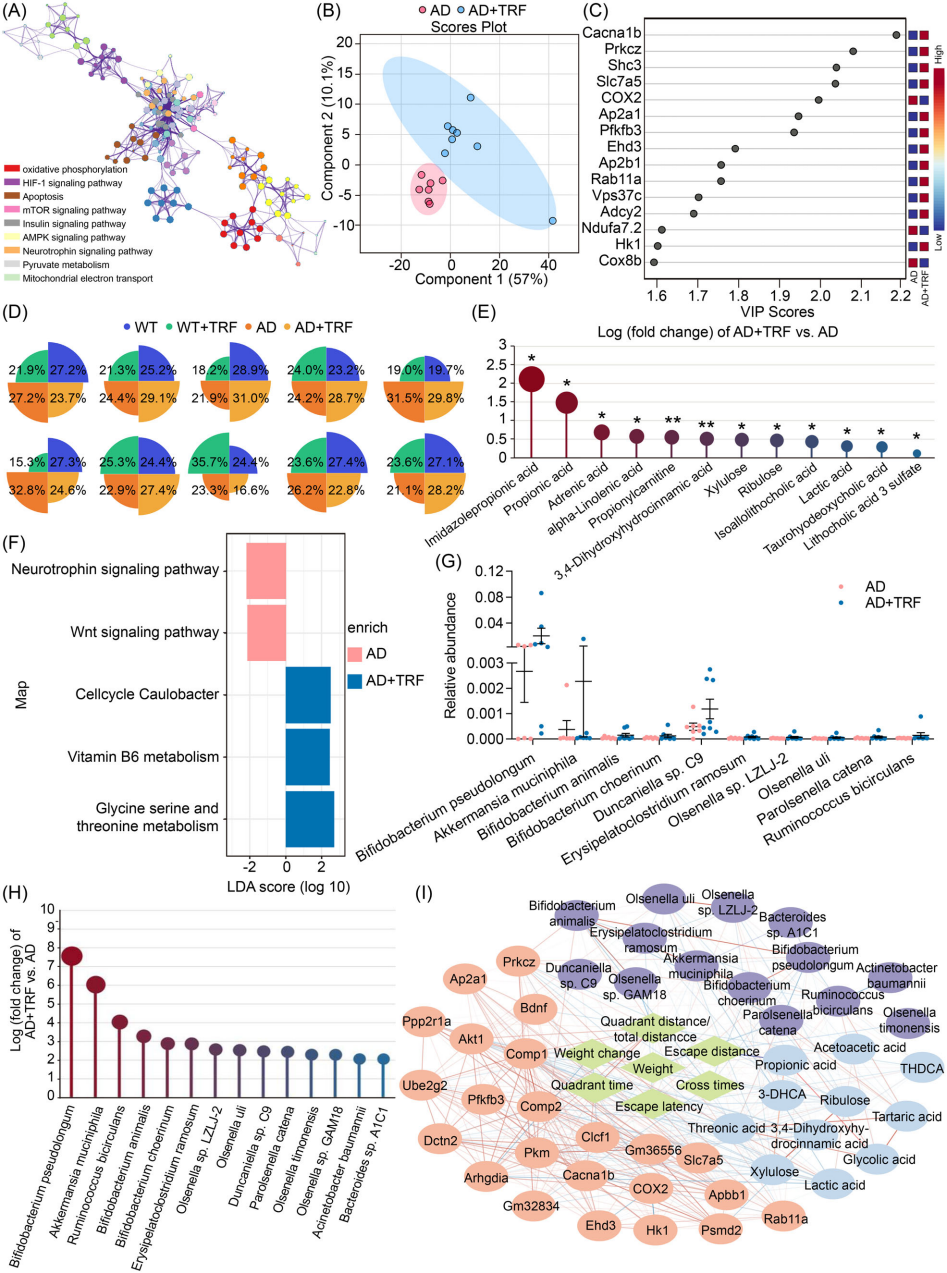
Integrated multi‐omics analysis revealing the gut microbiota‐metabolite‐brain axis underlying the TRF‐dependent effects on cognitive dysfunctions in AD mice. (A) Gene ontology terms of key genes that were identified by gene set enrichment analysis (GSEA) and were involved in the pathophysiology of AD (*n* = 7–10). (B) A great separation was achieved by performing the partial least squares discriminant analysis on key genes that were identified by GSEA and were involved in the pathophysiology of AD. (C) Partial least squares discriminant analysis (PLSDA) derived gene contributors for discriminating AD+TRF from AD group. (D) Summarized percentages of 10 main metabolite classes across 4 groups. (E) Fold change of top changed metabolites between AD+TRF versus AD. (F) Functional enrichment analysis on gut microbiome differed between AD+TRF and AD. (G) Relative abundances of key bacteria differed between AD+TRF and AD. (H) Fold change of top changed gut bacteria between AD+TRF versus AD. (I) Correlations of TRF‐regulated genes, metabolites, gut microbiota, biochemical parameters, and cognitive parameters.

Herein, we integrated hippocampal transcriptome, fecal metabolome, and gut microbiome to mine multimode evidence to decipher the microbiota‐metabolite‐brain axis underlying TRF‐dependent therapeutic effects on cognitive dysfunction. Specifically, we performed RNA sequencing on the hippocampi of mice. After mapping 183 Gb clean RNA‐SEQ reads of all mice against the *Mus musculus* genome, a total of 10,272 genes were detected, including 517 newly predicted genes lacking annotation. Gene set enrichment analysis (GSEA) was performed to clarify the biological functionality of genes differing between AD and AD+TRF (Table S1). Genes that were highly expressed after TRF were enriched in several biological processes, including IL‐6 signaling, the phosphatidic acid biosynthetic process, regulation of mitochondria, and positive regulation of behavior (Benjamini‐Hochberg adjusted *p* < 0.01), while genes involved in nervous system development, negative regulation of cellular process, and neurogenesis were downregulated by TRF (*p* < 0.05). Specific focus was given to the KEGG pathways profoundly involved in AD pathophysiology, such as neurotrophin signaling, mTOR signaling, apoptosis, AMP‐activated protein kinase (AMPK), and mitochondrial metabolism (Figure 2A), resulting in a total of 381 genes subjected to the partial least squares discriminant analysis (PLSDA). A clear discrimination between AD group and AD+TRF group was achieved (Figure 2B) and the core contributors identified by PLSDA for separation included *calcium voltage‐gated channel subunit alpha1 B* (*Cacna1b*), *protein kinase C zeta* (*Prkcz*), and *Cyclooxygenase‐2* (*COX2*), which were differentially expressed between the two groups as assessed by univariate analysis (Figure 2C).

Next, we performed a targeted‐metabolomics analysis of fecal samples to explore the alteration of fecal metabolism in TRF‐treated AD mice, which provided a functional readout of microbial activity. We quantified concentrations of 174 metabolites in fecal samples, including amino acids (*n* = 36), benzoic acids (*n* = 10), bile acids (*n* = 32), carbohydrates (*n* = 11), carnitines (*n* = 3), fatty acids (*n* = 32), imidazoles and indoles (*n* = 7), organic acids (*n* = 24), phenols and phenylpropanoic acids (*n* = 10), and SCFAs (*n* = 9) (Figure 2D and Table S2). TRF greatly affected the concentrations of bile acids and SCFAs in AD mice, by increasing 9.1% and 7.1% of their total concentrations, respectively, while reducing fatty acids and organic acids by 8.2% and 6.7% (Figure 2D). The pronounced increases induced by TRF were seen for imidazolepropionic acid (Fold change of AD+TRF vs. AD = 2.1, *p* = 0.04) and PA (Fold change of AD+TRF vs. AD = 1.5, *p* = 0.05) (Figure 2E). TRF also reduced the fecal levels of taurohyodeoxycholic acid, 3,4‐dihydroxyhydrocinnamic acid, propionylcarnitine, isoallolithocholic acid, lactic acid, xylulose, ribulose and lithocholic acid 3 sulfate (Figure 2E).

Intestinal microbes play a crucial role in ameliorating cognitive impairments. The gut microbiota composition was determined from mouse fecal samples using 16S rRNA gene sequencing and whole‐genome shotgun metagenomic sequencing, exhibiting consistent results. TRF failed to change microbiome diversity, metagenomic functional pathway richness, and metagenomic functional variation in AD mice, as determined by agnostic functional‐pathway testing (Figure 2F and Figure S3). However, TRF significantly influenced gut microbiota compositions, and the relative abundances of 84 microbial taxa identified by two sequencing technologies differed between AD and AD+TRF. Compared with AD mice, 36 microbial taxa were significantly upregulated and 48 were downregulated after TRF intervention (Figure S4A).

We then performed a trend clustering analysis by fuzzy C‐means algorithm to identify patterns of bio‐functional parameters, differentially expressed genes involved in AD pathophysiology, gut microbiota, and fecal metabolites (number of variables = 6372) of WT and AD mice undergoing different interventions. Sixteen distinct clusters of trending patterns were determined (Figure S4B). Variables mainly upregulated by TRF in AD mice were summarized in cluster 1, cluster 4, cluster 5, cluster 14, and cluster 16. Oppositely, cluster 3, cluster 8, and cluster 10 included variables downregulated by TRF in AD mice. Notably, a large number of TRF‐altered bio‐functional parameters of cognitive dysfunction and genes related to AD pathophysiology were clustered with SCFAs (i.e., PA), certain bile acids, and gut bacteria (i.e., clusters 4 and 16) (Figure S4B). Among them, *Akkermansia muciniphila* and *B. pseudolongum* were the most abundant bacteria and were significantly enriched by TRF in AD mice (Figure 2G). TRF also increased *Bifidobacterium animalis* and *Bifidobacterium choerinum*, but their abundances were relatively lower than *B. pseudolongum* (Figure 2G,H). Strong links between TRF‐induced changes in bio‐functional parameters, differentially expressed genes involved in AD pathophysiology, gut microbiota, and fecal metabolites were observed (Figure 2I). Collectively, these findings obtained from the integrated multi‐omics analysis indicate the microbiota‐metabolite‐brain axis that may mediate the TRF‐dependent beneficial effects on cognitive dysfunction in AD mice.

### Gut microbiota is essential for the protective effects of TRF on cognitive dysfunction in AD mice

**Figure 3:**
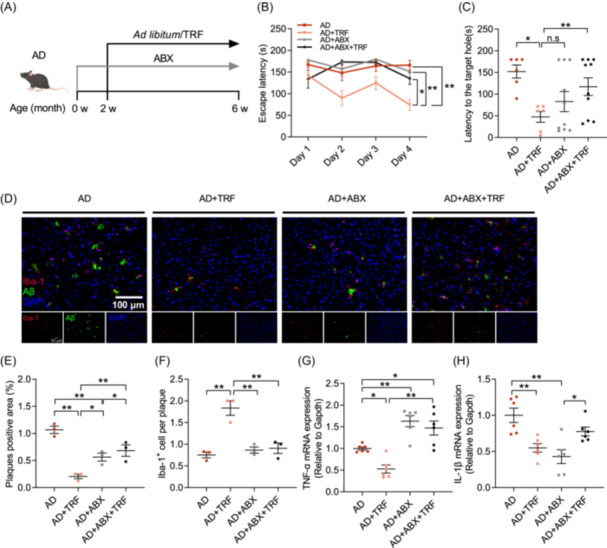
Gut microbiota removal eliminates the alleviation of TRF on AD mice. (A) Schematic of the treatment with broad‐spectrum antibiotics (ABX) and TRF in each group (*n* = 6–10). (B) Escape latency. (C) Latency to the target hole. (D) Aβ deposition (green) and Iba‐1^+^ (red) microglia immunohistochemical fluorescence images on mice cortex (*n* = 3) (Scale bar, 100 μm). (E) Quantification of plaques positive area. (F) Quantification of Aβ plaque‐associated microglia. (G) mRNA level of TNF‐α (*n* = 6). (H) mRNA level of IL‐1β (*n* = 6). Data are the means ± SEM. *p < 0.05, **p < 0.01; one‐way ANOVA with Tukey multiple comparisons test.

To further confirm the role of gut microbiota in mediating TRF‐dependent beneficial effects on cognitive dysfunction in AD mice, broad‐spectrum antibiotics (ABX) were used to deplete the original gut microbiota for 2 weeks, followed by another 4 weeks of TRF treatment. ABX treatment was implemented daily throughout the experiment to ensure the removal of the gut microbiota (Figure 3A). Depletion of gut microbiota abolished the ameliorating effect of TRF on cognitive impairment in AD mice, as evidenced by the escape latency, the latency to the target hole, and the number of approaches to the target hole assessed by the Barnes maze (Figure 3B,C and Figure S5D). In addition, compared with the AD+TRF group, antibiotic intervention alone did not significantly improve the cognitive ability of mice, as indicated by the escape latency (Figure 3B). The immunofluorescence staining showed that although ABX intervention significantly reduced Aβ deposition, the effect was not as strong as that of TRF treatment (Figure 3D,E and Figure S5F,G). Depletion of the gut microbiota eliminated the effect of TRF on improving AD pathological damage (Figure 3D–F and Figure S5F–H). In addition, the removal of gut microbiota also abolished TRF‐induced microglial recruitment to Aβ plaques (Figure 3D–F and Figure S5F–H). Antibiotic treatment exhibited distinct effects on the expression of neuroinflammatory factors in the brain, increasing the level of TNF‐α while reducing the level of IL‐1β (Figure 3G,H). However, with TRF intervention, both TNF‐α and IL‐1β were significantly reduced, and the effect was abolished upon gut microbiota removal (Figure 3G,H). The results demonstrate the essential role of gut microbiota in the protective effects of TRF on cognitive dysfunction in AD mice.

### Colonizing *B. pseudolongum*, alongside PA production, ameliorates cognitive dysfunction in AD mice

**Figure 4:**
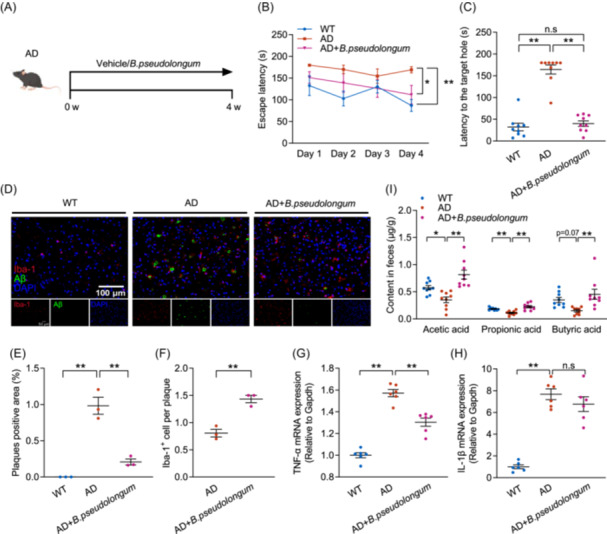
*Bifidobacterium pseudolongum* (*B.pseudolongum*) intervention mitigates cognitive dysfunction in AD mice. (A) Schematic of the treatment with *B.pseudolongum* or vehicle in each group (*n* = 9). (B) Escape latency. (C) Latency to the target hole. (D) Aβ deposition (green) and Iba‐1^+^ (red) microglia immunohistochemical fluorescence images on mice cortex (*n* = 3) (Scale bar, 100 μm). (E) Quantification of plaques positive area. (F) Quantification of Aβ plaque‐associated microglia. (G) mRNA level of TNF‐α (*n* = 6). (H) mRNA level of IL‐1β (*n* = 6). (I) Fecal levels of SCFAs (*n* = 9). Data are the means ± SEM. *p < 0.05, **p < 0.01; one‐way ANOVA with Tukey multiple comparisons test.

Considering the irreplaceable role of gut microbiota in mediating the alleviating effects of TRF on cognitive dysfunction in AD mice, and the strong associations between TRF‐induced significant enhancement in the abundance of *B. pseudolongum* and improved bio‐functional parameters involved in AD pathophysiology, we thus examined the effect of orally administering *B. pseudolongum* for 4 weeks on cognitive deficits in AD mice (Figure 4A). As expected, *B. pseudolongum* administration significantly declined the escape latency (Figure 4B), the latency to the target hole (Figure 4C), and the number of approaches to the target hole (Figure S6D), as well as reduced Aβ accumulation and increased Iba‐1^+^ cell surrounds per plaque in AD mice (Figure 4D–F and Figure S6F–H). In addition, *B. pseudolongum* administration also significantly declined the level of TNF‐α but had no effect on the reduction of IL‐1β (Figure 4G,H). In line with previous studies showing that *B. pseudolongum* promotes SCFAs production [20], we observed correlations between the relative abundance of *B. pseudolongum* and the fecal level of PA that were both increased by TRF. Noticeably, *B. pseudolongum* administration significantly restored fecal levels of acetic acid, PA, and butyric acid, which had evidently dropped in AD mice (Figure 4I). These results indicate that TRF induces *B. pseudolongum*, which is strongly associated with cognitive function and exhibits similar effects to TRF in alleviating cognitive impairment and pathological damage in AD, accompanied by SCFAs production.

### PA penetrates the blood‐brain barrier and improves cognition

**Figure 5:**
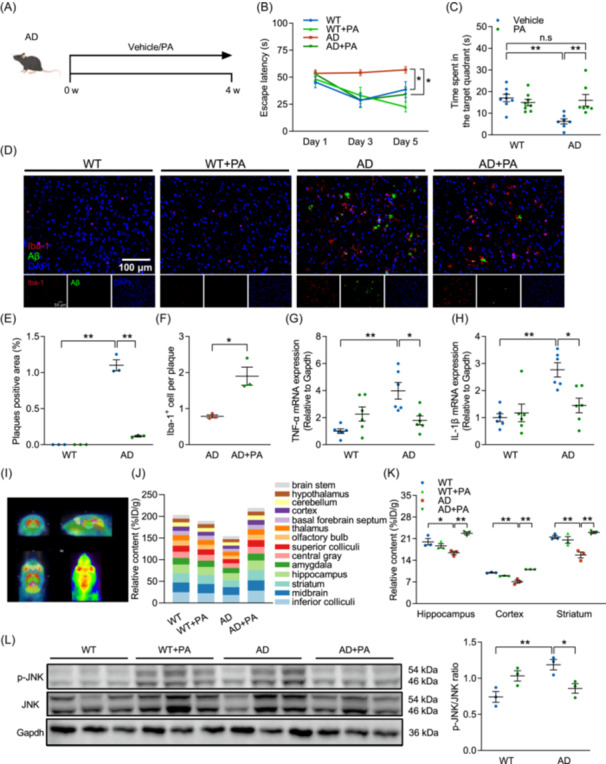
Propionic acid (PA) supplementation alleviates cognitive impairments in AD mice. (A) Schematic of the treatment with PA or vehicle in each group (*n* = 7–8). (B) Escape latency. (C) Time spent in the target quadrant. (D) Aβ deposition (green) and Iba‐1^+^ (red) microglia immunohistochemical fluorescence images on mice cortex (*n* = 3) (Scale bar, 100 μm). (E) Quantification of plaques positive area. (F) Quantification of Aβ plaque‐associated microglia. (G) mRNA level of TNF‐α (*n* = 6). (H) mRNA level of IL‐1β (*n* = 6). (I) Representative axial, sagittal, and coronal positron emission tomography (PET) images showing brain uptake with PA. (J) [^18^F]‐FPA level uptake (%ID/g) in the brain (*n* = 3). (K) PET quantification of [^18^F]‐FPA in hippocampus, cortex and striatum. (L) Western blots analysis of p‐JNK and JNK (*n* = 3). Data are the means ± SEM. *p < 0.05, **p < 0.01; two‐way ANOVA with Tukey multiple comparisons test.

To further confirm the importance of PA in the pathogenesis of AD and investigate the therapeutic effects of PA on cognitive deficits, sodium propionate was used to treat AD mice (Figure 5A). Intraperitoneal PA injection significantly enhanced cognitive function in AD mice (Figure 5B,C and Figure S7C), substantially reduced Aβ aggregation, promoted the recruitment of microglia to plaques, and increased BDNF intensity (Figure 5D–F and Figure S7E–I). TNF‐α and IL‐1β levels were also declined by PA treatment (Figure 5G,H). An assessment of PA penetration and metabolism was performed using positron emission tomography (PET) imaging in AD mice brains to determine whether PA penetrated the brain. PET imaging showed that PA could enter the brain through the blood‐brain barrier (Figure 5I), and PA metabolism mainly occurred in brain regions such as the inferior colliculi, midbrain, striatum, and hippocampus (Figure 5J,K). PET quantification confirmed that PA metabolic signals were significantly reduced in AD mice, especially in the hippocampus, cortex, and striatum; however, PA treatment significantly increased these signals (Figure 5K). SCFAs have been reported to exert anti‐neuroinflammatory effects through upregulating FFAR3 and suppressing the phosphorylation of nuclear factor kappa B (NF‐κB) and c‐Jun N‐terminal kinase (JNK) [21]. To investigate the impact of enhanced PA metabolism signals in the brain on neuroinflammation and Aβ deposition, the phosphorylation of NF‐κB and JNK was measured. It was revealed that PA intervention significantly reduced AD‐induced JNK phosphorylation but had no effect on NF‐κB phosphorylation (Figure 5L and Figure S7L), ultimately leading to the inhibition of β‐secretase (BACE1) expression (Figure S7K). Therefore, PA penetrates the blood‐brain barrier, restores PA metabolism disorder in the brain, inhibits JNK phosphorylation, and upregulates BDNF expression to alleviate AD‐induced cognitive damage and pathological progression.

### Knockdown FFAR3 abolishes the neuroprotective effect of TRF

**Figure 6:**
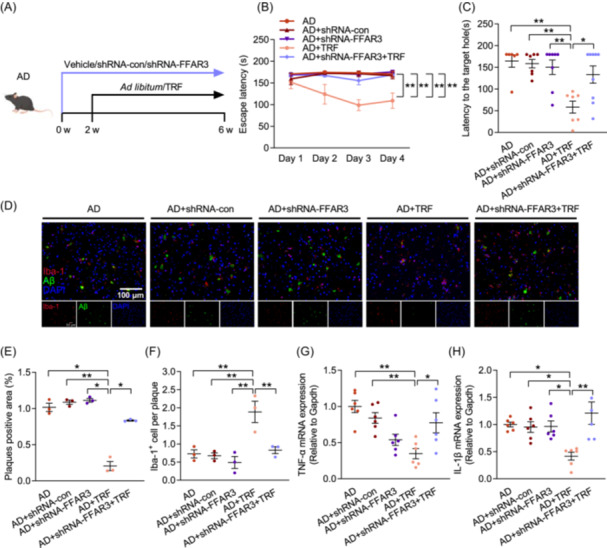
Free fatty acid receptor 3 (FFAR3) knockdown abolishes the benefits of TRF on AD mice. (A) Schematic of the treatment with shRNA‐FFAR3, shRNA‐con, and TRF in each group (*n* = 6–9). (B) Escape latency. (C) Latency to the target hole. (D) Aβ deposition (green) and Iba‐1^+^ (red) microglia immunohistochemical fluorescence images on mice cortex (*n* = 3) (Scale bar, 100 μm). (E) Quantification of plaques positive area. (F) Quantification of Aβ plaque‐associated microglia. (G) mRNA level of TNF‐α (*n* = 6). (H) mRNA level of IL‐1β (*n* = 6). Data are the means ± SEM. *p < 0.05, **p < 0.01; one‐way ANOVA with Tukey multiple comparisons test.

FFAR3 has been reported to combine with PA to regulate myocardial injury [22]. To observe the role of FFAR3 in the influence of TRF on AD, shRNA‐FFAR3 was injected into bilateral lateral ventricles of the brain to knockdown the mRNA level and protein expression of *FFAR3*, and shRNA‐con was injected as a viral vector control (Figure 6A and Figure S8A,B). Cognitive behavioral results showed that FFAR3 knockdown abolished the intervention effect of TRF compared to AD+TRF group (Figure 6B,C and Figure S8E). Furthermore, consistent with FFAR3 knockdown eliminating the protective effect of TRF on cognitive dysfunction, FFAR3 deprivation significantly reversed the decrease in Aβ over‐aggregation and reduced the recruitment of microglia to Aβ plaques observed with TRF treatment (Figure 6D–F and Figure S8G–I). Similarly, FFAR3 knockdown also abolished the inhibitory effect of TRF on the expression of neuroinflammatory factors (Figure 6G,H). The results indicate that FFAR3 is indispensable for the effects of TRF on Aβ clearance and cognition improvement.

### PA, as the biomarker of AD patients, plays a key role in alleviating cognitive impairments

**Figure 7:**
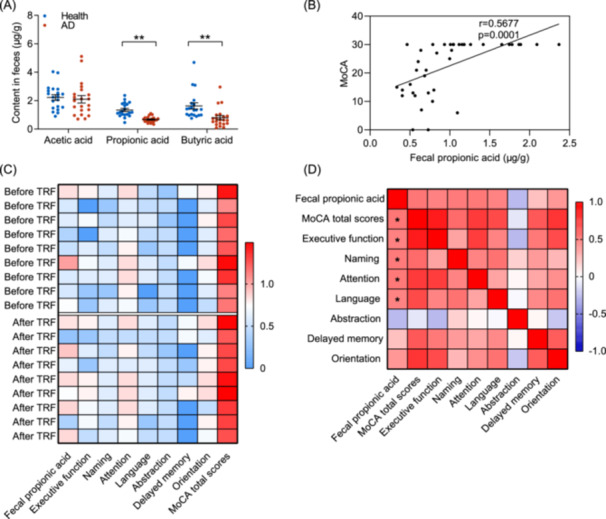
PA as the biomarker of AD patients plays a key role in alleviating cognitive impairments. (A) Fecal SCFAs levels of healthy and AD patients (*n* = 20‐21). (B) Scatter plot showing a strong positive correlation between MoCA score and fecal PA level (Pearson correlation coefficient *r* = 0.5677, *p* = 0.0001). (C) Heat map showing the concentration of PA in feces and MoCA score from AD patients receiving TRF treatment. (D) Heat maps of Spearman rank correlation between the altered fecal PA level and MoCA score. Data are analyzed using two‐tailed Student's *t*‐test, *p < 0.05, **p < 0.01.

To investigate whether SCFAs serve as a biomarker for AD patients, a case‐control study was conducted comparing fecal concentrations of PA and butyric acid between AD patients (*n* = 21) and healthy subjects (*n* = 20) (Table S4). Compared to healthy individuals, AD patients exhibited impaired cognitive function, as evidenced by significantly lower MoCA and mini‐mental state examination (MMSE) scores (Table S4). The results revealed significantly lower concentrations in AD patients (Figure 7A). The fecal concentration of PA exhibited a positive correlation with cognitive function, evaluated through MoCA and MMSE scores (Figure 7B and Figure S9A,B). Additionally, the fecal level of butyric acid displayed a significant positive association with cognitive function, albeit with a lower correlation coefficient compared to PA (Figure S9C,D).

To further clarify the role of TRF‐promoted PA in alleviating cognitive impairments of AD patients, fecal PA levels were detected in AD patients before and after the 4‐month TRF clinical intervention mentioned in Figure 1 above (Figure S9E). Significant correlations were observed between fecal PA, MoCA total scores, and specific cognitive domains (i.e., executive function, naming, attention and language) were recorded (Figure 7C,D). Through the cohort study and a small‐scale TRF clinical intervention trial, it is confirmed that fecal PA level is significantly positively correlated with cognitive status, suggesting that PA could serve as a potential biomarker for AD.

## DISCUSSION

Accumulating evidence has demonstrated the neuroprotective effects of TRF, primarily through animal studies. However, the mechanisms responsible for the neuroprotective effects of TRF on AD‐related cognitive impairments remain elusive. The present study reports that 4‐month TRF intervention greatly improved cognitive functions in AD patients and provides compelling evidence on the gut microbiota and SCFAs‐dependent effects of TRF intervention on ameliorating AD‐induced cognitive deficits, excessive Aβ accumulation, and neuroinflammation. TRF particularly elevated the fecal abundance of *B. pseudolongum* and PA. Supplementation of *B. pseudolongum* or its microbial metabolite PA effectively replicated the neuroprotective effects of TRF in AD mice.

Mechanistically, PA demonstrated the ability to cross the blood‐brain barrier and modulate brain pathology‐associated PA metabolism, thereby enhancing the cognitive function of AD mice by activating FFAR3, which regulated downstream signaling pathways, including JNK phosphorylation and BDNF expression. Notably, a significantly inverse association was found between fecal concentration of PA and cognitive dysfunction in AD patients, further underscoring PA as a key factor in TRF mitigating cognitive impairment. Our findings unequivocally demonstrate the microbiota (*B. pseudolongum*)‐metabolite (PA)‐ FFAR3 axis, which underlies the cognitive benefits of TRF, and highlight the translational potentials of nondrug intervention strategy, including TRF and other gut microbiota‐derived nutritional intervention, for halting AD progression.

Previous research has shown that maintaining TRF intervention for 6 months improved the cognitive status of the elderly [23]. Although the statistical power was limited by the sample size, our results proved that 4‐month TRF enhanced cognitive abilities in AD patients (Figure 1). Recent studies have shown a neuroprotective effect of TRF through upregulating BDNF level, reducing the expressions of inflammatory factors (IL‐6 and TNF‐α), and promoting neurogenesis [24, 25]. Previous studies have shown that IF and calorie restriction have inhibitory effects on AD‐like pathology, reflected in reduced lipoprotein lipase expression, decreased the levels of Aβ_40_ and Aβ_42_, and diminished the level of phospho‐tau [26, 27]. The latest research explained the improvement effect of TRF on AD cognition and Aβ from the perspective of circadian rhythm, focusing on restoring the disorder of brain transcriptional process through TRF intervention to affect AD‐induced sleep disturbance [12]. Although the mechanism and influence of TRF on cognitive impairment and neurological damage are controversial, the present study certified that TRF enhanced spatial memory ability, decreased Aβ deposition, promoted the recruitment of microglia to plaques, and increased BDNF level in AD mice (Figure 1). PLSDA of the hippocampal transcriptome effectively distinguished the gene contributors between the AD+TRF and AD groups, including *Cacna1b*, *Prkcz*, and *COX2*. TRF remarkably increased *Cacna1b* and *Prkcz* expressions while decreasing *COX2* expression (Figure S2). Cacna1b has been reported to be involved in maintaining synaptic signaling, with its dysregulation linked to the pathogenesis of AD [28]. Activation of protein kinase C, encoded by Prkcz, regulates the activity of BACE1 and controls the cleavage of the Aβ precursor APP [29]. Additionally, COX2 has been found to mediate the reciprocal regulation between IL‐1β and Aβ, promoting the pathogenesis of AD [30]. These findings indicate that TRF has beneficial effects on the development of neurodegenerative diseases. Specifically, TRF‐induced differentially expressed genes may serve as potential targets for the treatment of AD.

The gut microbiota‐brain axis gains worldwide recognition due to its important role in metabolism and neurodegenerative disorders. Noticeably, colonizing germ‐free mice with feces from AD patients and AD model mice decreased adult hippocampal neurogenesis, exacerbated memory impairment, and aggravated the development of AD [3, 31]. Interventions targeting the gut microbiota, such as antibiotic therapies, fecal microbiota transplants, and probiotic administrations, have demonstrated potential in mitigating the progression of AD [32]. Gut microbiota‐depletion by antibiotics has been shown to reduce Aβ deposition in the brain of AD mice yet increase soluble Aβ levels [33, 34]. Our findings indicate consistent results that the elimination of gut microbiota alleviates Aβ deposition. However, ABX intervention abolished the beneficial effects of TRF on AD‐induced cognitive dysfunction (Figure 3), which may be explained by the fact that ABX intervention removes both harmful gut microbiota associated with AD and the beneficial gut microbiota induced by TRF.

The effects of IF on gut microbiota have been extensively studied and documented [17]. Our previous study has shown that alternate‐day fasting increases *Lactobacillus* abundance, leading to cognitive enhancement, glial overactivation inhibition, and mitochondrial biogenesis augmentation [13]. Alternate‐day fasting exerts neuroprotective effects in multiple sclerosis model mice via elevating gut bacteria richness and promoting abundances of *Lactobacillaceae*, *Bacteroidaceae*, and *Prevotellaceae*, thereafter influencing microbial metabolic pathways in relation to antioxidative responses [35]. In AD, dysbiosis of gut microbiota is characterized by an imbalance with increased pro‐inflammatory and decreased anti‐inflammatory phyla [5]. Previous studies have reported significant differences in the composition and diversity of fecal microbiota between healthy individuals and AD patients, focused mainly on the alternation of *Ruminococcaceae*, *Clostridiaceae*, *Akkermansia*, and *Butyricicoccus* [36, 37, 38]. However, changes in the abundance of *Bifidobacterium* in the feces of AD patients are currently controversial. Some studies have suggested that an increase in *Bifidobacterium* is related to the pathological process of AD, while others have emphasized that a decrease in *Bifidobacterium* may be due to differences in the gut microbiota composition between healthy and those with AD patients [36, 37, 39]. These differences could be influenced by enrollment conditions and dietary environment of AD patients. However, some clinical intervention trials have demonstrated the cognitive benefits of *B. bifidum BGN4* and *B. longum BORI* in elderly individuals with cognitive dysfunction and depressive disorders [40]. Consistent with our findings, TRF greatly inhibited the decline of *Bifidobacterium*, particularly *B. pseudolongum*, in AD mice, and oral administration of B*. pseudolongum* improved cognitive function and mitigated pathological symptoms of AD (Figure 4). These controversies highlight the need for further investigation into the direct effects of *Bifidobacterium* and its derived metabolites on AD models and patients.

Our study also highlighted correlations between *B. pseudolongum* and fecal SCFAs, which were significantly enhanced by TRF in AD mice (Figure 2). Supplementation with SCFAs has been demonstrated to promote neural development, enhance cognitive functions, and ameliorate behavioral disorders [41, 42]. The combination of SCFAs and FFAR3 suppressed ERK/JNK/NF‐κB signaling, promoting recovery from cognitive deficits and AD‐type pathology [21, 43]. Our study further demonstrated that PA intervention effectively inhibited AD‐induced JNK phosphorylation, decreased BACE1 expression, mitigated Aβ aggregation, and attenuated neuroinflammatory responses (Figure 5). This underscores the direct impact of PA in preventing the development of neurodegenerative diseases. Specifically, regulation of the JNK pathway decreased Aβ deposition and levels of Aβ_40_ and Aβ_42_, which are known to mediate pathological cell death associated with AD [44]. Although mechanisms underlying the beneficial effects of PA on cognitive functions remain unclear, a recent clinical study has indicated that increased PA concentration in the cerebrospinal fluid of multiple sclerosis patients may enhance Treg cell mitochondrial function and morphology [45]. Moreover, lymphoblastoid cells from autism spectrum disorder patients have shown increased ability to use PA as fuel for energy production, although concentration‐dependent effects on mitochondrial function may lead to excessive reactive oxygen species (ROS) production and impaired energy production [46]. Based on our findings, PA metabolism was impaired in the brain of AD mice, indicating insufficient PA for energy consumption. PA supplementation effectively recovered brain utilization of PA (Figure 5). Collectively, the neuroprotective effect of TRF may be partly attributed to restoring energy metabolism pathway involving PA and the inhibition of JNK hyperphosphorylation, which may affect downstream signaling pathways. However, further investigation is warranted to explore the effects of PA on the mitochondria quality control system and respiratory enzymes in the brain.

PA enters cells by passive diffusion or via carrier proteins, such as SCFAs receptors FFAR2 and FFAR3 [22, 47]. Research indicates that FFAR3 is considered the only receptor for PA in the enteric nervous system and is expressed in human brain endothelial cells, whereas FFAR2 has not been detected in the central nervous system or peripheral nervous system [48, 49, 50]. PA has been reported to interact with FFAR3 to protect against myocardial ischemia‐reperfusion injury via reducing oxidative stress [22]. However, the impact of PA binding to FFAR3 in the brain on cognitive function remains largely unexplored. In this study, we validated that TRF ameliorated AD‐induced cognitive impairments through PA‐FFAR3 pathway using a FFAR3 AAV (Figure 6). Aligning with our study, PA has been found to exert a neuroregenerative role in the peripheral nervous system by binding to FFAR3 in a rat model of inflammatory neuropathies [51]. Additionally, we observed that PA intervention significantly inhibited the phosphorylation of JNK (Figure 5), a downstream signaling molecule of FFAR3, indicating that PA suppressed JNK phosphorylation via binding to FFAR3. TRF intervention reduced the levels of downstream neuroinflammatory factors (TNF‐α and IL‐1β) regulated by NF‐κB, an effect that was eliminated after knocking down FFAR3 (Figure 6). The expression and function of FFAR3 in different glial cells are unclear, more research is needed to explore the inhibitory effect of FFAR3 on neuroinflammatory responses in different glial cells.

A recent cohort study of patients with mild cognitive impairment and Parkinson's disease found that fecal SCFAs were significantly reduced, and the reduction of fecal SCFAs was negatively correlated with Aβ deposition in cognitive‐related brain regions, suggesting that SCFAs could serve as early diagnostic biomarkers to distinguish healthy individuals from those with neurodegenerative diseases [52]. Recent research supported similar findings, showing that the concentrations of PA and butyric acid in fecal samples inversely correlate with Aβ_42_ expression in individuals with mild cognitive impairment [53]. We also observed lower PA levels in the feces of AD patients who underwent TRF compared to controls, which were negatively correlated with cognitive status (Figure 7), supporting that PA may serve as a key regulator of TRF in mitigating cognitive impairment, as reflected by a significant correlation with MoCA scores (Figure 7).

The strength of our study lies in the extensive physiological analyses and multi‐omics interrogations conducted on mouse models, corroborated by human trials, providing novel insights into the mechanisms underlying the cognition improvement of TRF and identifying key intestinal contributors, *B. pseudolongum* and PA. However, our study has several limitations. The effects of PA on the nervous system are controversial. Excessive or acute PA interventions, such as doses over 200 mg/kg, have been associated with autism and propionic acidemia [54, 55]. The inconsistencies in findings may be attributed to disparities in dosages and administration methods used across different interventions, crucially underscoring the importance of dosage and administration methods in determining the efficacy of PA. Herein, mice received intraperitoneal injection of 0.1 g/kg body weight of sodium propionate, resulting in the improved cognition in AD mice and delayed the progression of AD pathology in mice (Figure 5). Moreover, TRF has been reported to improve cognition via multiple mechanisms. For instance, prolonged fasting induces the production of ketone bodies. Ketone supplementation has demonstrated beneficial effects on executive function, memory, and language abilities in individuals with mild cognitive impairment [56]. Additionally, apart from PA, other SCFAs, such as acetic acid and butyric acid, have shown cognitive benefits. Acetic acid has been shown to alleviate cognitive impairment in type 1 diabetic mice, and butyrate ameliorates cognitive deficits in AD mouse models via binding to its receptors FFAR2 and FFAR3 [57, 58]. However, neither acetic acid nor butyric acid changed significantly after TRF in our study, and no significant association was observed between acetic acid and cognitive parameters in AD patients. Lastly, the small‐scale TRF population clinical intervention trial we conducted had a limited sample size, and factors such as gender and genetics were not considered. Nevertheless, it provides valuable insight and serves as a useful reference for designing larger‐scale TRF clinical intervention studies in the future. Additionally, it is of great interest to collect samples from patients with different neurodegenerative diseases to determine whether reduced fecal PA levels are specific for AD or common across neurodegenerative disorders. Biochemical analyses of cerebrospinal fluid are also warranted. Large‐sample studies on the effects of PA, butyric acid, and their related gut microbiota on AD patients are necessary in the future.

## CONCLUSION

In summary, our findings support that TRF ameliorates cognitive deficits by modulating the *B. pseudolongum*‐PA‐FFAR3 axis in AD mice. A 4‐month clinical intervention of TRF with 16 h of fasting per day alleviated cognitive impairment in AD patients. The beneficial effects of TRF on cognitive decline in AD may stem from an enhanced relative abundance of *B. pseudolongum* and augmented PA, ultimately suppressing neuroinflammation and Aβ deposition in the brain, in the gut microbiota‐dependent manner. Knockdown of SCFAs receptor FFAR3 abrogated the positive impact of TRF on mitigating cognitive dysfunction. Furthermore, a significant association between PA and cognition status was demonstrated in a case‐control study. Overall, our findings open up new avenues for non‐pharmaceutical intervention targeting gut microbiota and its metabolites, especially *Bifidobacteria* and PA, for neurodegenerative diseases.

## METHODS

### Animal experiments

The male transgenic mice with 5xFAD mutations (Stock No: 006554) maintained on C57BL/6J background were purchased from the Jackson Laboratory (Bar Harbor, ME). The 5xFAD mice express five Alzheimer's disease (AD)‐associated mutations on human amyloid precursor protein and the human presenilin 1 (PSEN1), which includes the Swedish (K670N/M671L), Florida (I716V), and London (V717I) mutations in APP, and the M146L and L286V mutations in PSEN1. The method used for generating 5xFAD mice has been described previously [42]. The 5xFAD mice were periodically backcrossed with female C57BL/6J stock (Stock No: 006554) from Jackson Labs. To identify the genotype of the generations, mice were measured for both the APP and PS1 transgenes with polymerase chain reaction (PCR) using DNA samples obtained from ear tissue (DNA extraction Kit (centrifugal column), Beijing Betek Biotechnology Co. Ltd).

The present study included five animal experiments. According to Management Measures for Laboratory Animals of Northwest A&F University, ethical approval for animal experiments was obtained, with the assigned approval number XN2023‐0610. All experiments conducted in this study strictly adhered to the applicable ethical standards and regulations governing animal research.

All mice were fed with a standard chow diet and housed individually at a constant temperature of 22 ± 2°C under a relative humidity of 55 ± 5% and a 12/12 h light–dark cycles with lights on at 8:00 a.m. (ZT0) in the facility of Northwest A&F University. Animal experiments examined behavioral tests and pathological analyses comprehensively. Brain tissue from the hippocampus was macro‐dissected and stored at −80°C until analysis. Detailed protocols of animal experiments are shown below:

(1) A 12‐week intervention was conducted to investigate the effect of TRF on cognitive impairment in AD mice. The 6‐month‐old male 5xFAD (AD) mice and their age‐matched wild‐type (WT) mice on the same background were randomly grouped into either the ad libitum or TRF group (*n* = 7–10 per group). Mice undergoing TRF intervention were fed for 8 h per day during 11 p.m. to 7 a.m. (ZT15‐ZT23) with unrestricted access to water. Fecal samples were collected at the end of interventions for metabolome and microbiome profiling.
(2) To demonstrate the irreplaceable role of gut microbiota in mediating the TRF‐induced alleviating benefits on cognitive impairment in AD mice, we compared the behavioral and pathological performance of AD mice after a 4‐week TRF intervention in the presence or absence of gut microbiota. The TRF regimen schedule was the same as previously mentioned. For gut microbial depletion, the 7‐month‐old male 5xFAD mice were orally administered an antibiotic cocktail (ABX) for gut microbial depletion. ABX contained 1 g/L neomycin sulfate, 1 g/L ampicillin sulfate, 1 g/L metronidazole, and 0.5 g/L vancomycin hydrochloride (Sigma‐Aldrich, Shanghai, China), as previously reported [41]. ABX was added to the drinking water 2 weeks before the TRF regimen and was administered throughout the experiment to maintain the removal of the gut microbiota. AD mice were randomly assigned to either the *ad libitum* or TRF group (AD group, AD+TRF group, AD+ABX group, and AD+ABX+TRF group) (*n* = 6–10 per group). Microbiota depletion was confirmed by plating feces on BHI agar plates under anaerobic and aerobic conditions, and quantitative real‐time PCR (qRT‐PCR). qRT‐PCR was performed with the universal bacteria‐specific primer: (8F: AGAGTTTGATCCTGGCTCAG; 338R: CTGCTGCCTCCCGTAGGAGT).
(3) We investigated whether oral gavage of *Bifidobacterium pseudolongum* (*B. pseudolongum*) could alleviate cognitive impairment in AD mice. Before the administration, *B. pseudolongum* (ATCC 25526; zlzt, Wuhan, China) was anaerobically cultured at 37°C for 24 h, then harvested by centrifugation (5000 rpm, 5 min, 4°C) and resuspended in PBS with a concentration of 1 × 10^9^ colony‐forming units (CFU)/mL. The mice were randomly divided into three groups. The 7‐month‐old male AD mice were given oral gavage by a suspension of *B. pseudolongum* (100 μL/mouse) or PBS vehicle per day for 4 weeks (*n* = 9 per group). The dosage of *B. pseudolongum* in the current study was chosen according to previous experiments and related references [59, 60]. Fecal DNA was extracted from mice, quantified, and subjected to qRT‐qPCR amplification to determine the colonization status of *B. pseudolongum* (16S forward: 5′‐CGCCGATGATGGGATGCTTTACA‐3′ and 16S reverse 5′‐AGATCATCTAAACACCACCCACAC‐3′). Fecal samples were collected at the end of interventions for the colonization status of *B. pseudolongum* and SCFAs measurements.
(4) In the treatment with propionic acid (PA), the 7‐month‐old male AD mice were randomly divided into 4 groups (*n* = 7–8 per group). Mice were injected intraperitoneally with sodium propionate (0.1 g/kg body weight; Sigma‐Aldrich) dissolved in normal saline or normal saline vehicle per day for 4 weeks. The concentration of sodium propionate was chosen based on the previous literature [61, 62].
(5) To demonstrate the role of free fatty acid receptor 3 (FFAR3), a key receptor that combines with PA to regulate myocardial injury, in the TRF‐dependent effects on cognitive impairment in AD mice, we further investigated the neuroprotective effect of TRF in AD mice with FFAR3 knockdown [22]. Specifically, recombinant adeno‐associated virus serotype 2/9 (AAV2/9) vectors which carry a CMV promoter with GFP reporter (FFAR3‐shRNA, 5′‐TTTGCTAAACCTGACCATTTC‐3′) (Heyuan Biotechnology Co.) or GFP vector control (Heyuan Biotechnology Co.) to knockdown FFAR3 gene expression or as control [22]. Animals received i.c.v. injection of different treatments and were divided into five groups (7 months old, *n* = 6–9 per group): AD group (injection with PBS), AD+shRNA‐con group (injection with AAV2/9‐GFP), AD+shRNA‐FFAR3 group (injection with AAV2/9‐GFP‐FFAR3), AD+TRF group (injection with PBS) and AD+shRNA‐FFAR3+TRF group (injection with AAV2/9‐GFP‐FFAR3). The mice were anesthetized with 2% isoflurane, and then the AAV2/9 vector was randomly i.c.v. injected into lateral ventricles on both sides of each mouse through stereotaxic apparatus (68025; RWD, Shenzhen, China) (coordinates from Bregma in mm: AP‐0.3, ML+1.0, DV‐2.5 and AP‐0.3, ML‐1.0, DV‐2.5) at a virus dose of 1 × 10^12^ as reported [63, 64]. Infusion of 1 μL virus solution was controlled at 200 nL/min using a microinjection pump (LEGATO 130; KD Scientific Inc). After injection, the injection needle was maintained in place for an additional 10 min to prevent reflux. After the mice recovered for 1 week, TRF intervention was performed for 4 weeks.

### The case‐control study

To assess associations between fecal levels of SCFAs and AD symptoms in participants, we recruited from 30 healthy people and 30 AD patients from Peking University Shenzhen Hospital during October 2023 and June 2024. According to the “DSM‐IV” judgment method for AD, we screened out the population with dementia caused by AD (excluding dementia caused by surgery or other reasons). Participants in the present study were excluded if they (1) had a history of inflammatory bowel disease, carbohydrate malabsorption, hormonal imbalance, known allergy to food additives, or any other serious disease; (2) had a history of gallbladder removal, gastrointestinal tract, and cranial surgery; (3) had a parasitic infection; (4) had suicidal thoughts, attempts or aggressive behavior; (5) used drugs known to affect gastrointestinal function, blood pressure and blood lipids, hormone supplements, allergy/asthma drugs, proton pump inhibition (6) took probiotics or antibiotics or prebiotics (dietary fiber, oligosaccharides) within 8 weeks before the test; (7) abused alcohol or drugs; (8) experienced cognitive dysfunction or had a history of major trauma; (9) had a history of cerebrovascular disease or cognitive dysfunction caused by brain tumors and other primary brain lesions; (10) smoking addiction.

Participants undergo the following assessments: (1) MoCA and MMSE to evaluate comprehensive cognitive function. (2) A medical interview to confirm medical history and current medication use. (3) The analysis of the four cerebrospinal fluid components (including Aβ_40_, Aβ_42_, total‐Tau, and p‐Tau, voluntary participation), to serve as the diagnostic basis for physicians in diagnosing AD. (4) PET‐computed tomography (PET‐CT) scans (encompassing Aβ‐PET and Tau‐PET, voluntary participation), as a diagnostic reference for physicians in diagnosing AD; (5) The analysis of AD blood biomarkers (including Aβ_40_, Aβ_42_, total‐Tau, and p‐Tau and others, voluntary participation), to further assist physicians in diagnosing AD. Based on the screening requirements and following the diagnostic criteria of the NINCDS‐ADRDA, a total of 20 healthy subjects (MoCA ≥ 27 and MMSE ≥ 27) and 21 AD patients (MoCA < 27 and MMSE < 27, individuals with abnormalities in the cerebrospinal fluid analysis, PET‐CT scans, or blood biomarkers) were selected into the cohort (Table S4). All participants were provided with a detailed explanation of the research protocol and obtained written informed consent. The study received approval from the Ethics Committee of Peking University Shenzhen Hospital under approval number No. 2023‐108.

### TRF clinical trial

Participants were recruited between October 2023 and June 2024, and data collection was completed in March 2024. The inclusion criteria for AD patients were the same as above. Overall, 10 AD patients (two males and eight females, aged from 58 to 81 years old) were diagnosed by cerebrospinal fluid analysis, PET‐CT scans, or blood biomarkers and enrolled in the TRF intervention study. One week before the TRF intervention, all participants received diet and lifestyle guidance, delivered through expert lectures and printed promotional materials. This guidance was grounded in the Dietary Guidelines for Chinese Residents 2016 and the Chinese National Healthy Lifestyle Action 2017–2025. Participants were instructed to eat *ad libitum* 8 h a day, with the time selection can be 8:00 a.m.–16:00 p.m. or 9:00 a.m.–17:00 p.m. or 10:00 a.m.–18:00 p.m., for 4 months. Participants were not allowed to consume calorie‐containing foods or beverages (except black coffee or unsweetened tea) during the fasting period and were encouraged to drink adequate water throughout the intervention period. Cognitive scale (MoCA and MMSE), serum, and feces samples were collected at baseline and intervention endpoints. Adverse events were recorded at each follow‐up. The clinical nutritionist initially determined the level of the adverse events based on the situation and recommend whether to continue the intervention. Adverse events complained by the subjects during the entire intervention process were recorded and reported to the project team in real time by the researchers. Adverse events required a follow‐up visit within 3 days to confirm whether there has been improvement. If there was no improvement, the project clinical expert group would decide whether to terminate the subject's intervention process. At the end of the TRF intervention, 9 participants completed (two males and seven females, aged from 58 to 81 years old), and 1 participant dropped out because she could not stick to TRF. All participants were provided with a detailed explanation of the research protocol and obtained written informed consent. The study received approval from the Ethics Committee of Peking University Shenzhen Hospital under approval number No. 2023‐108 and registered on the Chinese Clinical Trial Registry (ChiCTR2400092653).

### Morris water maze test

The Morris water maze was used to study the functional evaluation of brain region related to spatial learning and memory, performed as previously described with minor modifications [13]. The task was conducted in a circular tank (diameter: 150 cm and height: 35 cm) filled with opaque water (23°C–25°C). A 4.5‐cm‐diameter and 14.5‐cm‐height platform was placed at the center of northeast quadrant of the pool. The pool was divided into four quadrants of equal area, with four prominent visual cues on each side of the four quadrants of the pool. This test includes two periods: initial spatial training and probe test. On the first day, all mice were trained to stand on the platform for 60 s to realize the existence of the hidden platform and memorize the environment. During the following 3‐d initial spatial training, the hidden platform trials were used to evaluate spatial learning ability, with white‐dyed water (Food grade titanium dioxide) and the platform submerged 0.5–1.0 cm below the water surface. On each day, the mouse was placed into the water maze at one of the four quadrants and allowed to swim freely until they found and climbed onto the platform. If the mice failed to escape within 60 s, they were gently conducted to the platform and allowed to stay there for 30 s. Each mouse was subjected to four trials per day, and the starting position was different for each trial. Escape latency (s) was recorded by video‐tracking system. To assess the spatial retention of the location of the hidden platform, a probe trial was conducted 24 h after the last acquisition session. On the 4th day, the platform was removed from the pool, and each mouse was allowed one 60 s swim probe trial. Finally, the data of the time spent in the target quadrant and the number of platform crossings were recorded by the photosensors and analyzed using a computerized video‐tracking system (Super Maze software, Shanghai Xinruan Information Technology Co. Ltd).

### Barnes maze test

Spatial learning and memory were examined with the Barnes maze as previously described [65]. Briefly, the maze consisted of a brightly illuminated circular platform (92 cm diameter), with 20 evenly distributed holes located around. The experiment was conducted over a 5‐day period, with each mouse trained on ability to locate the escape box once per day during Days 1–4. One of the holes is set to place the escape box at the bottom of the target hole, and the other holes are empty. Each mouse was placed under an opaque start box in the center of the maze for 5 s and explore the platform freely after the start box was lifted. The trial finished when the mouse had climbed into the escape box or after 3 min had exhausted. The escape latency and primary latency were recorded. On Day 5, a probe test was conducted, the escape box was removed, and mice were placed in the center of the maze in which they were free to navigate for 3 min. The maze and escape box were wiped with 70% ethanol after each trial, and the maze was rotated. Percentage time in the correct quadrant was determined using the Smart 3.0 tracking software (Super Maze software, Shanghai Xinruan Information Technology Co. Ltd).

### Real time‐qPCR

To quantify the mRNA expression levels of the regulation genes of neurotrophic factors and inflammatory cytokines and protein, total RNA from brain tissues was isolated by Trizol reagent (Invitrogen) according to the manufacture's instruction. Up to 5 μg of total RNAs isolated from tissues were reverse‐transcribed by using PrimeScriptTM RT Master Mix reverse transcription kit (TaKaRa PrimeScript RT Master Mix) and the CFX96T real‐time system (Bio‐Rad). Gene‐specific mouse primers were used, as mentioned in Table S3. Ct values were normalized to GAPDH, and the relative gene expression was calculated with the 2^−△△Ct^ method.

### Western blots

Western blot analysis was detected as described [13]. Proteins from 30 mg liver tissues of 4 mice/group were collected using ice‐cold radio‐immunoprecipitation assay (RIPA, P0013C, Beyotime Institute of Biotechnology) buffer containing 1 mM PMSF and protease inhibitors (P1005, Beyotime Institute of Biotechnology). The denatured protein samples were loaded onto 10% SDS PAGE gels for electrophoresis and then transferred onto 0.45 μm polyvinylidene fluoride (PVDF) membranes. After blocking the binding sites for 2 h with 5% dried skim milk at 37°C, the membranes were incubated overnight at 4°C with the appropriate primary antibodies, including FFAR3 (1:1000, ER64606; HuaBio), Gapdh (1:1000, Lot#5174S; Cell Signaling Technology), JNK (1:1000, sc‐7345; Santa Cruz), p‐JNK (1:500, sc‐6254; Santa Cruz), NFκB (1:1000, sc‐8008; Santa Cruz), p‐ NFκB (1:1000, sc‐136548; Santa Cruz). The next day, the membranes were washed with TBST and incubated at room temperature for 2 h with the corresponding secondary antibody. Protein expression was measured by chemiluminescence (Bio‐Rad Laboratories), and the blots were quantified with Image J.

### Immunofluorescence staining

The procedure of immunofluorescence (IF) staining was performed according to a preceding study [13]. Fixed brain sections were immersed in xylene and gradient ethanol for dewaxing and rehydration. After that, paraffin sections were quenched with 3% peroxide for 10 min to block endogenous peroxidase. The goat serum sealed the sections at room temperature for 20 min, and then the primary antibody was incubated at 4°C overnight, including amyloid‐beta (1:1000, SIG‐39320; Biolegend), Iba‐1 (1:1000, Cat# ab178847; Abcam), BDNF (1:500, Cat# ab108319; Abcam). On the second day, after PBS washing, the tissue slices were incubated with a secondary antibody (Cy3 conjugated Goat Anti‐Rabbit IgG (H+L), Servicebio) for 20 min. Rinse in PBS eight times, and histologic sections have sealed the tablet with sealant containing DAPI. Co‐staining of amyloid‐beta and Iba‐1 was to wash the primary antibody on the second day and then incubate the corresponding secondary antibody. After washing, the tissue slices were incubated with another primary antibody overnight. On the third day, the tissue slices were washed with the primary antibody and incubated the secondary antibody before washing and sealing the slide. The sections were observed under a fluorescence microscope and photographed (Olympus). The positive area was measured by Image J as well.

### SCFAs levels analysis

The SCFAs in the feces were measured by GC, as previously described [42]. Briefly, feces (180–200 mg) was mixed with 1 mL MilliQ water and vortexed at room temperature for 10 min. The samples were added 150 μL of 50% H_2_SO_4_ (*w/w*) and fully oscillated. Then, 1.6 mL of diethyl ether was added and incubated on ice for 20 min. After that, the supernatant was obtained by centrifugation (8000 rpm, 5 min, 4°C) and filtrated by 0.2 µm filters (Tianjin Branch billion Lung Experimental Equipment Co., Ltd). It was transferred to a clear GC vial, and diethyl ether was used as the internal standard. The concentration of SCFAs was analyzed using GC‐2014C (Shimadzu Corporation, Kyoto, Japan), fitted with a DB‐FFAP capillary column (30 m × 0.25 µm × 0.25 mm) (Agilent Technologies). A standard curve was established with different concentrations of a standard mixture containing acetate (A116173), propionate (P110445), and butyrate (B11se0438) (Aladdin Bio‐Chem Technology Co., Ltd). Peaks were integrated by using the Shimadzu GC solution software. All SCFAs concentration is expressed as µg/g in feces.

### Positron emission tomography imaging

[^18^F]‐Fluoropropionic acid ([^18^F]‐FPA) was synthesized from the precursor ethyl‐2‐bromopropionate, with [^18^F] fluorination and hydrolysis reaction described in detail as previous research [66]. The total decay‐corrected radiochemical yields of [^18^F]‐FPA were over 40%, with a specific activity of around 50 GBq/μM. The radiochemical purities of [^18^F]‐FPA were over 98%. PET imaging was performed with [^18^F]‐FPA using a MadicLab Box 071 (Shandong Madic Technology Co., Ltd). The mice were intravenously injected with 11.7 ± 5.2 MBq of [^18^F]‐FPA and scanned 30 min afterward. Before undergoing PET, the animals were anesthetized with 2.5% isoflurane, moved to the heated scanner bed, and kept anesthetized with 1.5%–2% isoflurane during the scan. The duration of the scan was 90 min. PET data were reconstructed using two‐dimensional ordered‐subsets expectation maximization and corrected for dead time and radioactive decay. All subsequent processing of the PET images and the radioactivity distribution in different brain regions was performed using PMOD, version 4.3 (PMOD Technologies LLC).

### RNA sequence analysis

Total RNA was extracted using TriZol (Invitrogen) according to the manufacturer's instructions, followed by being qualified and quantified using a NanoDrop and Agilent 2100 bioanalyzer (Thermo Fisher Scientific). RNA sequencing libraries were prepared using BGISEQ‐500 (BGI‐Shenzhen). Sequencing data were filtered and trimmed using Trimmomatic v0.38 (Bolger, Lohse, & Usadel, 2014) to obtain high‐quality clean‐read data. Clean reads were mapped to the *Mus musculus* genome sequence (ftp://ftp.ncbi.nlm.nih.gov/genomes/all/GCF/000/001/635/GCF_000001635.26_GRCm38.p6) using Hisat2 v2‐ 2.1.0. The reads of each sample were then assembled into transcripts and compared with reference gene models using StringTie v1.3.4d. We merged the 35 transcripts to obtain a consensus transcript using a StringTie‐Merge program. Transcripts that did not exist in the CDS database of the *Mus musculus* genome were extracted to predict new genes. The gene expression FPKM values were calculated using the StringTie Merge program based on the consensus transcript. KEGG and Gene Ontology enrichment analyses were performed using the WebGestalt 2019 (WEB‐based Gene Set Analysis Toolkit) and were visualized using Metascape (http://metascape.org/).

### Metabolomics

Fecal samples were subjected to the targeted quantitative metabolomics profiling designed to cover gut microbiota‐derived and related metabolites using an ultra‐performance liquid chromatography coupled to tandem mass spectrometry (UPLC‐MS/MS) system (ACQUITY UPLC‐Xevo TQ‐S, Waters Corp.). In brief, the lyophilized fecal sample vortexed vigorously with ice methanol containing internal standards, and the supernatant was obtained. Then, ice‐cold 50% methanol solution was added to dilute the sample and stored at −20°C for 20 min, followed by 4000*g* centrifugation. The supernatant mixed with internal standards for each sample was sealed before UPLC‐MS/MS profiling. The instrument parameters were set as follows: C18 analytical column (2.1 × 100 mm, 1.7 μM); column temperature 40°C; mobile phases A (water with 0.1% formic acid), mobile phases B (acetonitrile: IPA, 70:30). The quality control samples were made up of pooled samples and were run every 14 samples. Raw data generated by UPLC‐MS/MS were processed using the MassLynx software (v4.1, Waters) for peak integration, calibration, and quantification for each metabolite. A total of 174 metabolites were quantitatively detected, mainly including amino acids, fatty acids, and bile acid (Table S2). Sample preparation and metabolomics profiling were conducted by Metabo‐Profile Biotechnology (Shanghai) Co., Ltd) [67].

### 16S rRNA gene sequencing and metagenomic sequencing

We applied both 16S rRNA gene sequencing and whole genome shotgun metagenomics to quantify the gut microbiome composition of fecal samples, which were conducted by BGI‐Shenzhen China National GeneBank. In brief, fecal samples were collected in a clean environment, and total cellular DNA was extracted with the E.Z.N.A. Stool DNA Kit (Omega) according to the company's instructions. The bacterial hypervariable V3‐V4 region of 16S rRNA was chosen for MiSeq (Illumina) paired‐end 300 bp amplicon analysis using primers 341_F (5′‐ CCTACGGGNGGCWGCAG‐3′) and 802_R (5′‐TACNVGGGTATCTAATCC‐3′). Library preparation followed a published method 9. The raw reads were merged and trimmed, chimeras were removed, and zero‐radius Operational Taxonomic Units (zOTUs) with UNOISE were implemented in Search (v2.6.0). The green genes (13.8) 16S rRNA gene database was used as a reference for annotation. Rarified OTU data were used to predict functional genes with PICRUSt (v1.1.3). The predicted genes were annotated with KEGG at different levels, and the significantly abundant pathways were identified by edgeR with FDR‐*p* < 0.1.

The taxonomic analysis method of metagenomics was described previously [68]. The clean reads were mapped to the Kraken database (k2_pluspfp_20220908) by Kraken2 v2.1.2, a k‐mer‐based tool, to generate a taxonomic profile. The results of Kraken2 were processed with Bracken v2.5. Relative abundance in a sample is defined as the number of assigned reads compared to the number of total clean reads. The R package “vegan” was used to evaluate the alpha and beta diversity, specifically, the Shannon index and Bray‐Curtis dissimilarity based on the relative abundance profiles were calculated by the function “diversity” and “vegdist” [69]. Functional annotation was performed with the HUMAnN3 v3.5 for functional analysis of the metagenomes of each sample. The abundance of the kegg‐orthology was determined using the HUMAnN3 tool based on the official database released in 201901. The 16S rRNA gene sequencing and whole genome short gun metagenomics were conducted by BGI Genomics Co., Ltd.

### Data analyses

Data were reported as means ± SEM of at least three independent experiments. The significance of the observed differences was assessed using two‐tail unpaired Student's *t*‐test (for two groups), or two paired Student's *t*‐test (for before and after TRF clinical intervention), or one‐way ANOVA (for three or five groups), or two‐way ANOVA (for four groups), followed by Tukey post hoc test by GraphPad Prism 8.0 software. Significance was set at *p* < 0.05 and expressed as *p < 0.05 and **p < 0.01 in Figures. Data analysis was performed in a blinded manner.

Statistics for OMICs data were conducted by R programming (Version 4.1). For transcriptomics data, we performed GSEA to clarify the biological functionality differed between AD and AD+TRF (R packages ClusterProfile). The significantly abundant pathways were identified by *p* < 0.05. Specific focus was given to the KEGG pathways profoundly involved in the pathophysiology of AD, that is, neurotrophin signaling, mTOR signaling, apoptosis, AMPK, and mitochondrial metabolism (Table S1), and a total of 381 genes were then subjected to the partial least squares discriminant analysis (PLSDA, R packages mixOmics).

A Wilcoxon test was used to examine the influence of TRF on the number of microbial species in AD mice. Chao1 and ACE index reflected community richness, and the Shannon and Simpson index reflected community diversity. FDR‐*p* < 0.05 was considered as a significant difference. The Fuzzy C‐means clustering technology (R packages ClusterGVis) was conducted to integrate TRF‐induced changes in bio‐functional parameters, differentially expressed genes involved in AD pathophysiology, microbial taxa, and fecal metabolites. This method is one of the typical clustering algorithms in data mining and has been widely used to identify the changing patterns in high throughput and high dimensionality data induced by intervention.

## AUTHOR CONTRIBUTIONS

**Yihang Zhao**: Methodology; validation; writing—review and editing; writing—original draft; investigation; software; formal analysis; data curation; visualization; conceptualization; project administration; supervision. **Mengzhen Jia**: Conceptualization; investigation; writing—original draft; methodology; validation; visualization; software; data curation; supervision; formal analysis; project administration. **Chen Ding**: Methodology; validation; visualization; investigation; data curation; writing—original draft; project administration; software. **Bingkun Bao**: Investigation; methodology; validation; visualization; project administration; formal analysis; data curation. **Hangqi Li**: Methodology; validation; visualization; investigation; project administration; formal analysis; writing—original draft. **Jiabin ma**: methodology; validation; investigation; formal analysis; project administration; visualization. **Weixuan Dong**: Methodology; software; data curation; supervision; resources; formal analysis. **Rui Gao**: Methodology; software; data curation; resources; supervision. **Xuhui Chen**: Methodology; investigation; formal analysis; supervision; data curation. **Jiao Chen**: Data curation; methodology; investigation; formal analysis; supervision. **Xiaoshuang Dai**: Software; data curation; formal analysis; project administration. **Yuanqiang Zou**: Software; data curation; formal analysis. **Jun Hu**: Funding acquisition; conceptualization; methodology; validation; investigation; supervision; resources; formal analysis; project administration. **Lin Shi**: Conceptualization; methodology; software; data curation; writing—original draft; writing—review and editing; supervision; investigation; validation; formal analysis. **Xuebo Liu**: Conceptualization; methodology; funding acquisition; project administration; formal analysis; resources; supervision; investigation; validation; data curation; visualization. **Zhigang Liu**: Conceptualization; investigation; funding acquisition; methodology; validation; formal analysis; supervision; project administration; resources; writing—original draft; writing—review and editing; visualization; data curation; software.

## CONFLICT OF INTEREST STATEMENT

The authors declare no conflicts of interest.

## ETHICS STATEMENT

According to Management Measures for Laboratory Animals of Northwest A&F University, ethical approval for animal experiments was obtained with the assigned approval number XN2023‐0610. The case‐control study and TRF clinical trial received approval from the Ethics Committee of Peking University Shenzhen Hospital under approval number No. 2023‐108 and registered on the Chinese Clinical Trial Registry (ChiCTR2400092653).

## Supporting information


**Figure S1.** Time‐restricted feeding (TRF) alleviates cognitive impairments in Alzheimer's disease (AD).
**Figure S2.** Relative abundance of differential genes.
**Figure S3.** The effects of TRF on gut microbiota composition of AD mice.
**Figure S4.** The identify TRF‐induced changing patterns of bio‐functional parameters, differentially expressed genes involved in AD pathophysiology, gut microbiota, and fecal metabolites.
**Figure S5.** Gut microbiota mediates neuroprotective influence of TRF.
**Figure S6.** *Bifidobacterium pseudolongum* (*B. pseudolongum*) alleviates cognitive impairments in AD mice.
**Figure S7.** Propionic acid (PA) intervention mitigates AD‐induced cognitive impairment.
**Figure S8.** Knockdown of free fatty acid receptor 3 (FFAR3) eliminates the improved effect of TRF.
**Figure S9.** A case‐control study of fecal SCFAs in AD patients and a clinical intervention study of TRF on AD patients.

**Table S1.** Gene set enrichment analysis (GSEA) of AD vs. AD+TRF group.
**Table S2.** The levels of 174 metabolites in AD group and AD+TRF group.
**Table S3.** Primer sequences used for semi‐quantitative RT‐qPCR analysis.
**Table S4.** Demographics and clinical parameters of subjects in the healthy and AD groups.

## Data Availability

Data deposition: 16S rRNA gene sequence data is available on NCBI BioProject accession nos. PRJNA1014591 (https://www.ncbi.nlm.nih.gov/bioproject/PRJNA1014591). Transcriptome and metagenome sequence data have been deposited in the Genome Sequence Archive under accession number CRA022584 and CRA022537 (https://bigd.big.ac.cn/gsa/browse/CRA022584 and https://bigd.big.ac.cn/gsa/browse/CRA022537). Original data have been deposited in figshare data set as follows: https://doi.org/10.6084/m9.figshare.26129467.v1. Supplementary materials (figures, tables, graphical abstract, slides, videos, Chinese translated version, and update materials) may be found in the online DOI or iMeta Science http://www.imeta.science/.
