# Supplementary material for: Time‐restricted feeding mitigates Alzheimer's disease‐associated cognitive impairments via a B. pseudolongum‐propionic acid‐FFAR3 axis
Source: Imeta. 2025 Feb 21;4(2):e70006. doi: 10.1002/imt2.70006 (PMC11995186; doi:10.1002/imt2.70006)
Supplement: Supplementary file 1 — Figure S1. Time‐restricted feeding (TRF) alleviates cognitive impairments in Alzheimer's disease (AD). Figure S2. Relative abundance of differential genes. Figure S3. The effects of TRF on gut microbiota composition of AD mice. Figure S4. The identify TRF‐induced changing patterns of bio‐functional parameters, differentially expressed genes involved in AD pathophysiology, gut microbiota, and fecal metabolites. Figure S5. Gut microbiota mediates neuroprotective influence of TRF. Figure S6. Bifidobacterium pseudolongum (B. pseudolongum) alleviates cognitive impairments in AD mice. Figure S7. Propionic acid (PA) intervention mitigates AD‐induced cognitive impairment. Figure S8. Knockdown of free fatty acid receptor 3 (FFAR3) eliminates the improved effect of TRF. Figure S9. A case‐control study of fecal SCFAs in AD patients and a clinical intervention study of TRF on AD patients. [file IMT2-4-e70006-s001.docx]

Supporting information to

**Time-restricted feeding mitigates Alzheimer's disease-associated cognitive impairments via a *B. pseudolongum*-propionic acid-FFAR3 axis**

Yihang Zhao^1#^, Mengzhen Jia^1#^, Chen Ding^1^, Bingkun Bao^1^, Hangqi Li^1^, Jiabin Ma^1^, Weixuan Dong^3^, Rui Gao^3^, Xuhui Chen^4^, Jiao Chen^4^, Xiaoshuang Dai^6^, Yuanqiang Zou^7^, Jun Hu^4*^, Lin Shi^5*^, Xuebo Liu^1*^, Zhigang Liu^1,2*^

^1^ College of Food Science and Engineering, Northwest A&F University, Yangling, 712100, China

^2^ Northwest A&F University Shenzhen Research Institute, Shenzhen, 518000, China

^3^ The First Affiliated Hospital of Xi’an Jiaotong University, Xi’an, 710061, China

^4^ Peking University Shenzhen Hospital, Shenzhen, 518004, China

^5^ Shaanxi Normal University, Xi’an, 710119, China

^6^ Xbiome, Shenzhen, 518083, China

^7^ BGI Research, Shenzhen, 518083, China

^#^These authors contributed equally: Yihang Zhao, Mengzhen Jia

^*^Correspondence: [zhigangliu@nwsuaf.edu.cn](mailto:zhigangliu@nwsuaf.edu.cn) (Zhigang Liu), [xueboliu@nwsuaf.edu.cn](mailto:xueboliu@nwsuaf.edu.cn) (Xuebo Liu), [linshi198808@snnu.edu.cn](mailto:linshi198808@snnu.edu.cn) (Lin Shi), [dochj@163.com](mailto:dochj@163.com) (Jun Hu)


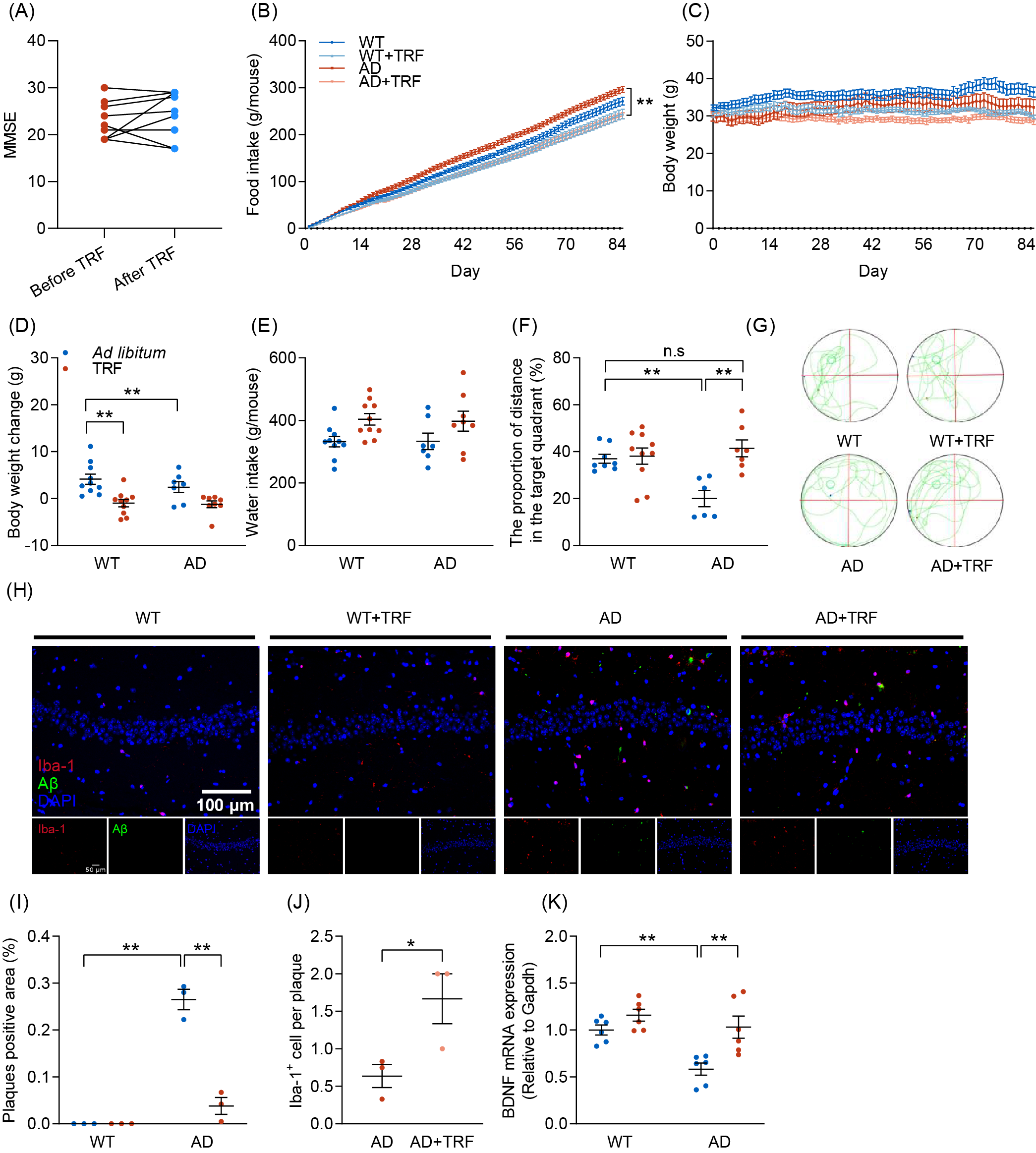
Figure S1 Time-restricted feeding (TRF) alleviates cognitive impairments in Alzheimer’s disease (AD). (A) Quantification of mini-mental state examination (MMSE) scores from AD patients before and after 4-month TRF intervention (*n* = 9), as assessed by two-tailed paired Student’s t-test. (B) Food intake (*n* = 6-10 per group). (C) Water intake. (D) Body weight. (E) Body weight change. (F) The proportion of distance in the target quadrant. (G), Mouse trajectory during the probe trial. (H) amyloid-beta (Aβ) deposition (green) and ionized calcium-binding adaptor molecule 1 (Iba-1^+^) (red) microglia immunohistochemical fluorescence images on mice hippocampal CA1 area (*n* = 3) (Scale bar, 100 μm.). (I) Quantification of plaques positive area. (J) Quantification of Aβ plaque-associated microglia. (K) mRNA level of brain-derived neurotrophic factor (BDNF) in the cortex (*n* = 6). Data are the means ± SEM. ^*^*p* < 0.05, ^**^*p* < 0.01; two-way ANOVA with Tukey multiple comparisons test.


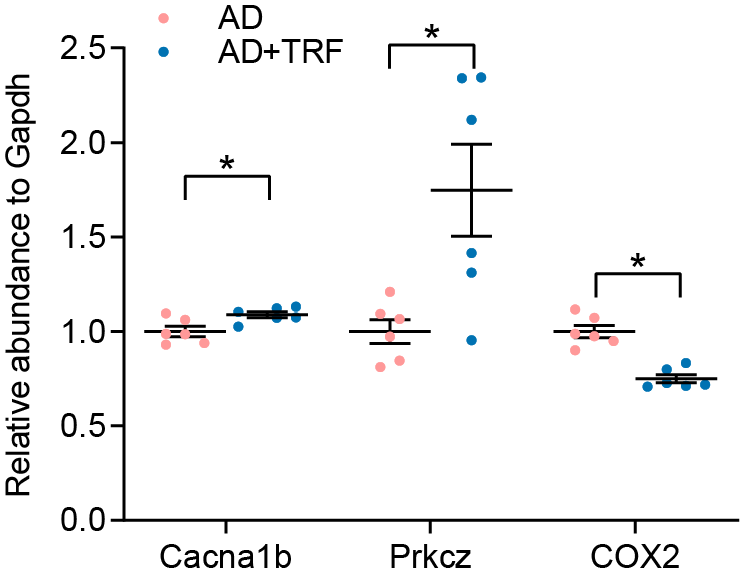
Figure S2 Relative abundance of differential genes. Relative abundance of *calcium voltage-gated channel subunit alpha1 B* (*Cacna1b*), *protein kinase C zeta* (*Prkcz*), and *Cyclooxygenase-2* (*COX2*), as assessed by two-tailed unpaired Student’s t-test (*n* = 6), ^*^*p* < 0.05, compared with AD mice.


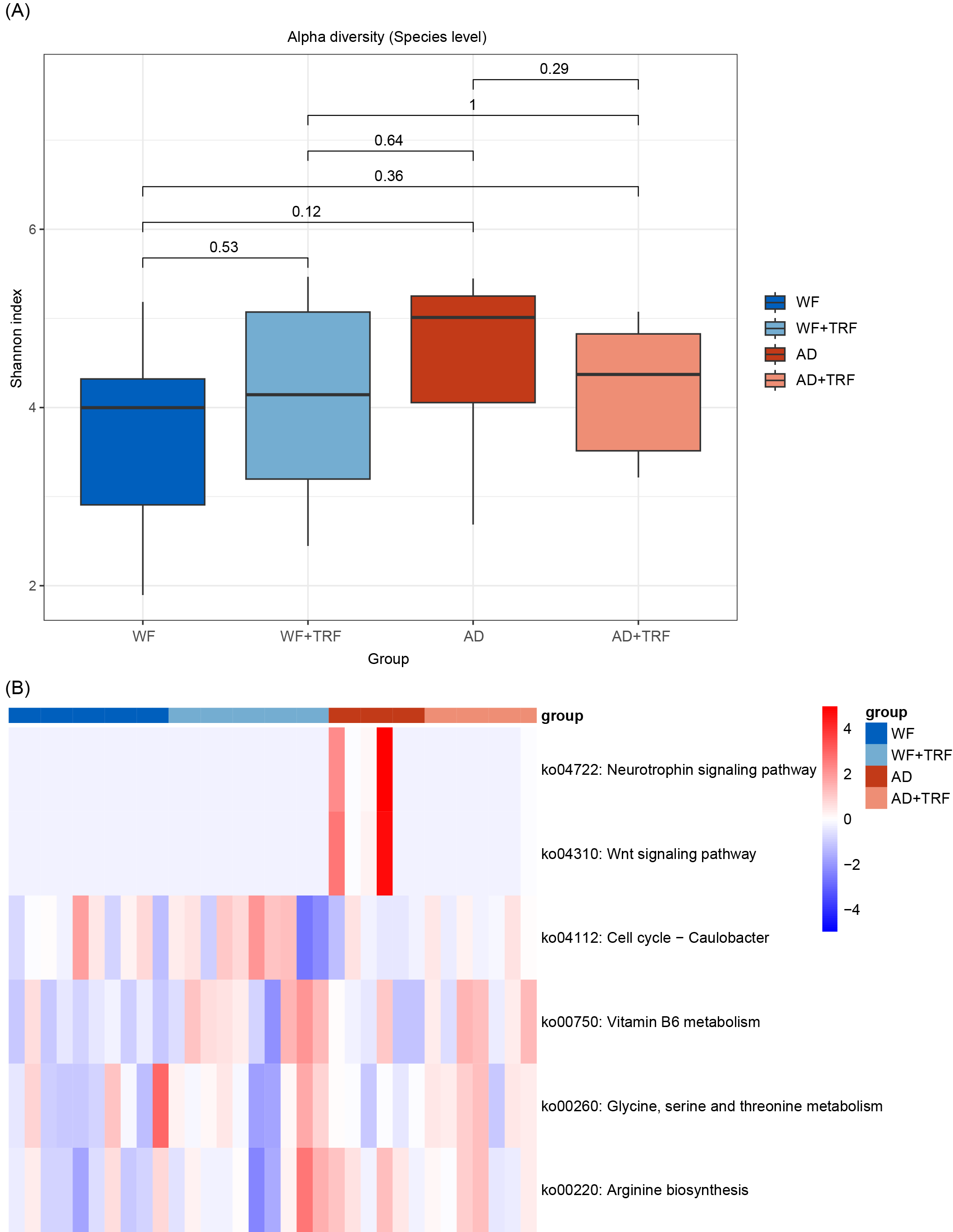
Figure S3 The effects of TRF on gut microbiota composition of AD mice. (A) Alpha diversity of species level between treatments (*n* = 6-10). (B) The microbial function pathways.


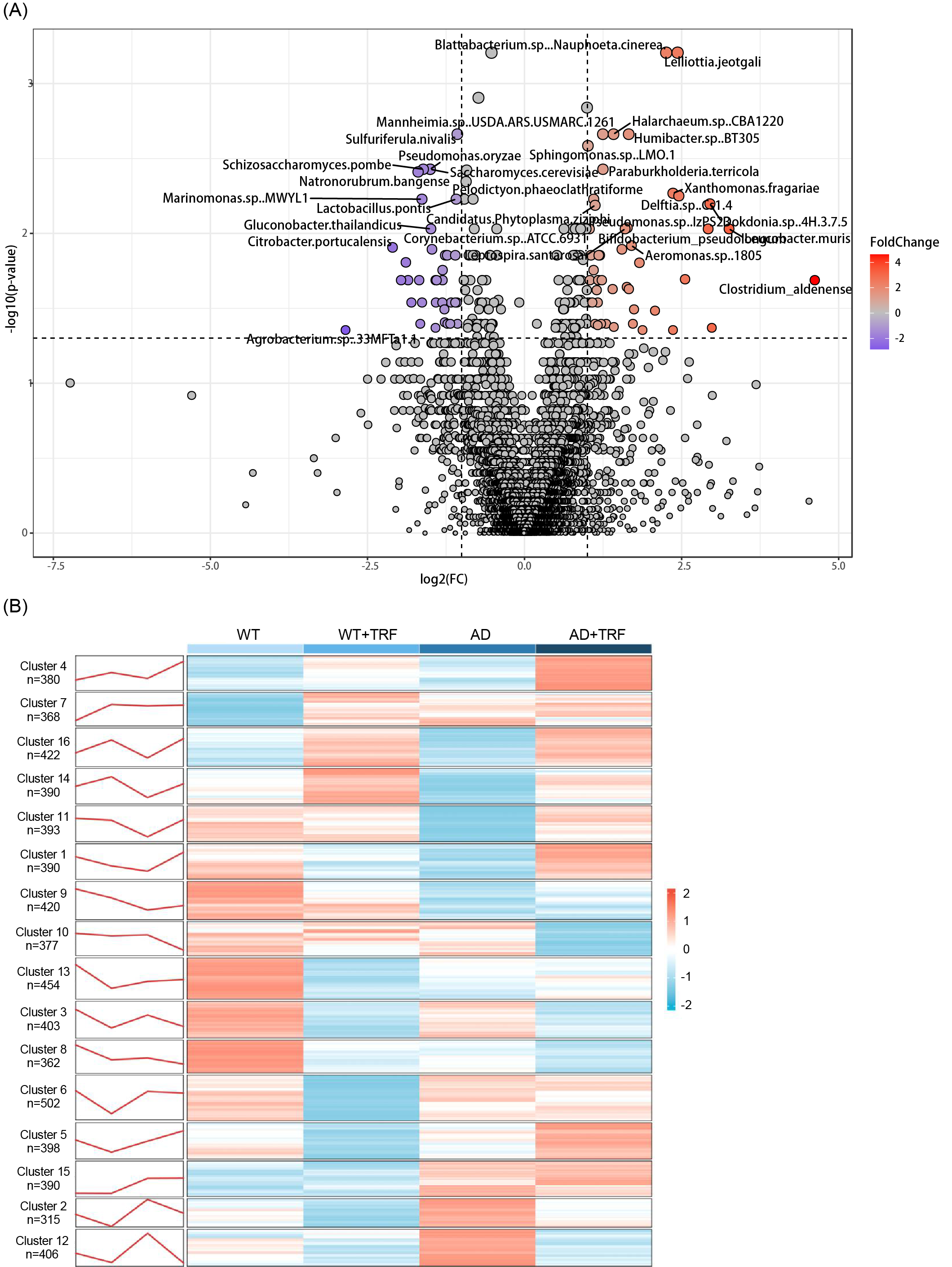
Figure S4 The identify TRF-induced changing patterns of bio-functional parameters, differentially expressed genes involved in AD pathophysiology, gut microbiota and fecal metabolites. (A) Volcano plot showing differences in the abundance of gut microbiota after TRF intervention, the colored (purple or orange) regions indicate statistically significant (*p* < 0.05) differences with a magnitude of at least 1.5-fold (*n* = 7-10). (B) Trend clustering analysis based on the fuzzy c-means algorithm was used to identify patterns of 6372 variables, reflecting how they could be altered by TRF in WT or AD mice.


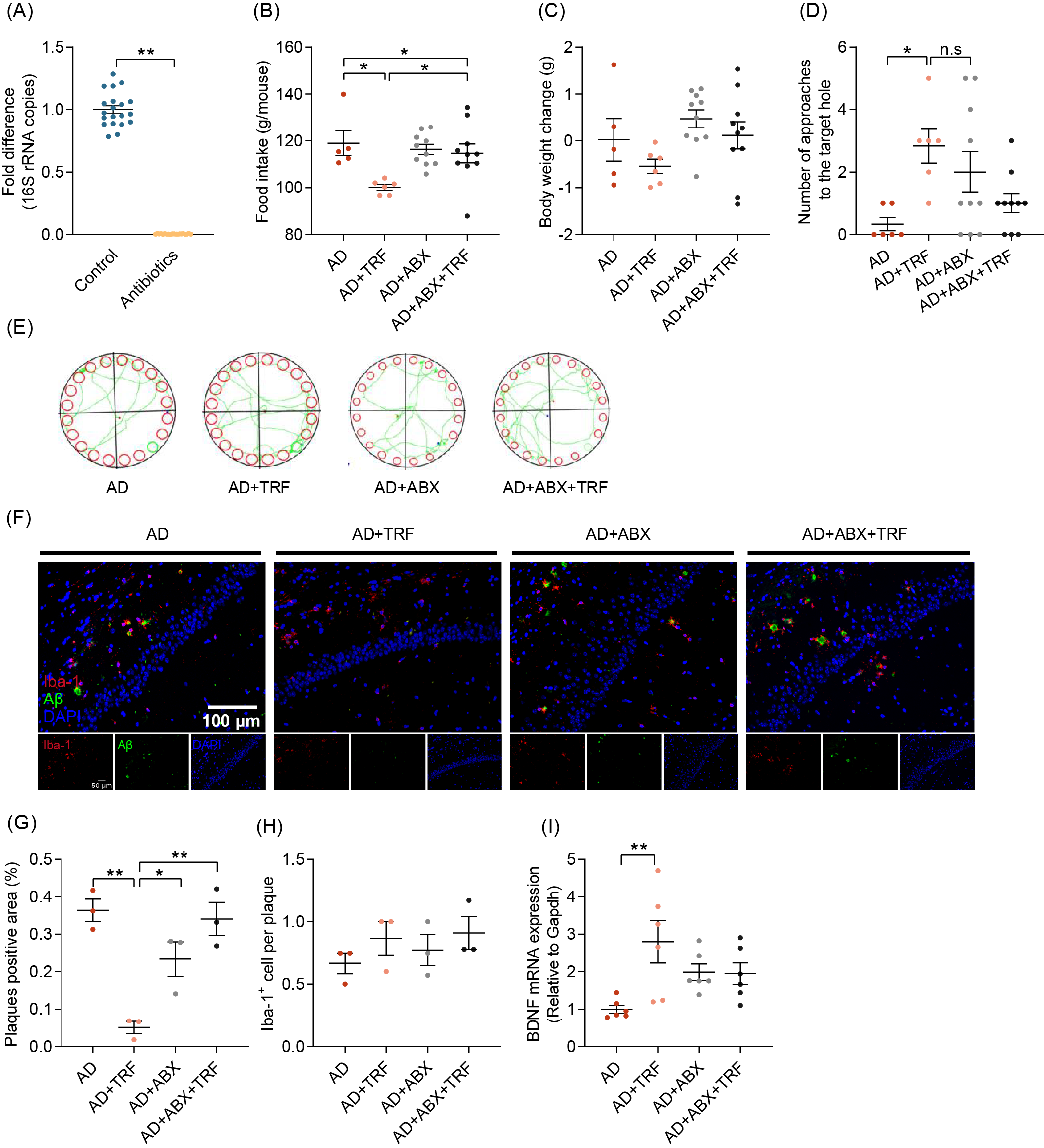
Figure S5 Gut microbiota mediates neuroprotective influence of TRF. (A) qPCR of 16S rRNA analysis to ensure the removal efficacy of microbiota (*n* = 20), as assessed by Student’s t-test, ^**^*p* < 0.01, compared with control mice fed with normal water. (B) Food intake (*n* = 6-10). (C) Body weight change. (D) Number of approaches to the target hole. (E), Mouse trajectory during the probe trial. (F) Aβ deposition (green) and Iba-1^+^ (red) microglia immunohistochemical fluorescence images on mice hippocampal CA1 area (*n* = 3) (Scale bar, 100 μm.). (G) Quantification of plaques positive area. (H) Quantification of Aβ plaque-associated microglia. (I) mRNA level of BDNF in the cortex (*n* = 6 per group). Data are the means ± SEM. ^*^*p* < 0.05, ^**^*p* < 0.01; one-way ANOVA with Tukey multiple comparisons test.


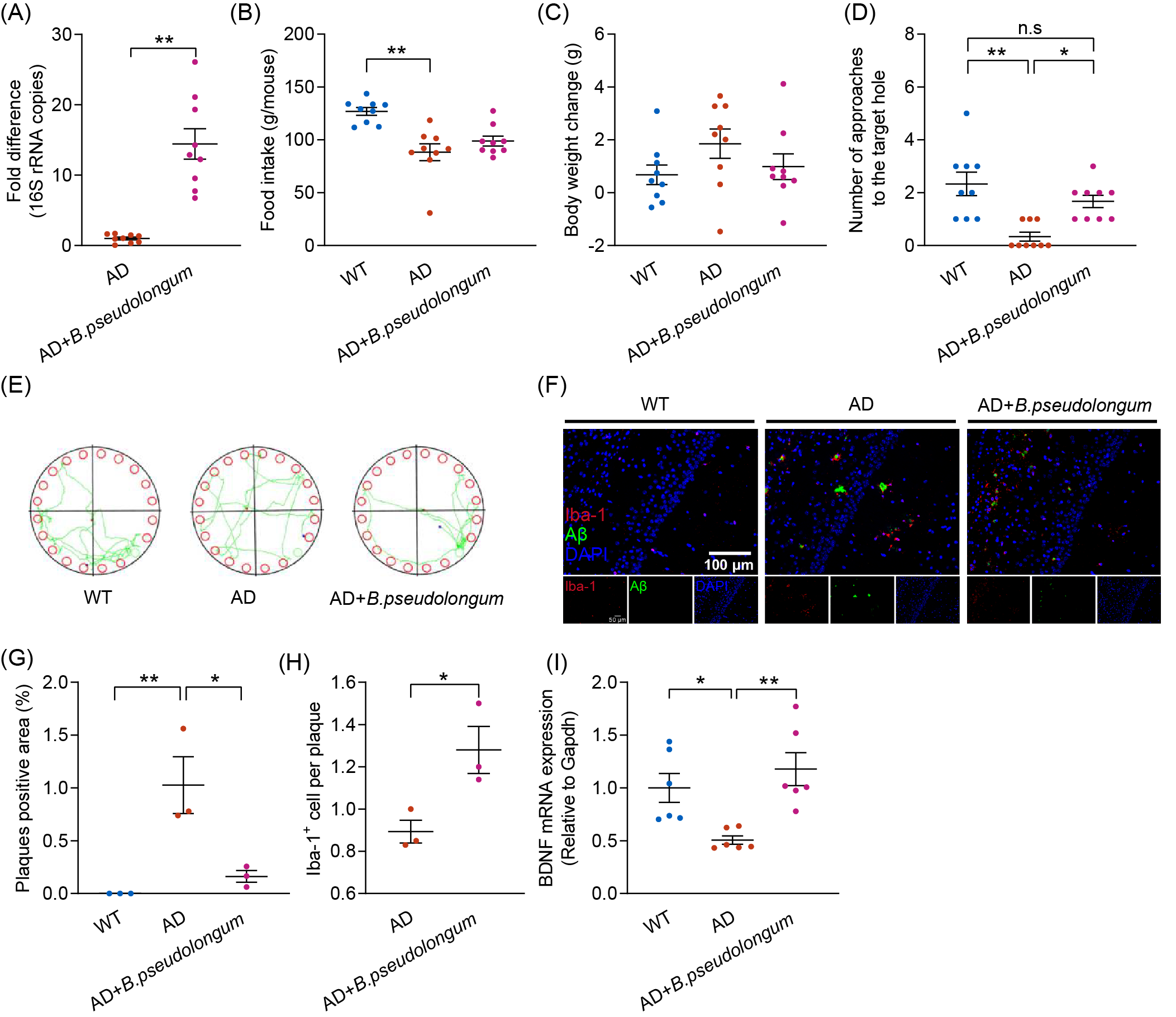
Figure S6 *Bifidobacterium pseudolongum* (*B. pseudolongum*) alleviates cognitive impairments in AD mice. (A) qPCR of 16S rRNA analysis to ensure the colonization with *B. pseudolongum* (*n* = 9), as assessed by Student’s t-test, ^**^*p* < 0.01, compared with control mice treatment with vehicle. (B) Food intake (*n* = 9). (C) Body weight change. (D) Number of approaches to the target hole. (E), Mouse trajectory during the probe trial. (F) Aβ deposition (green) and Iba-1^+^ (red) microglia immunohistochemical fluorescence images on mice hippocampal CA1 area (*n* = 3) (Scale bar, 100 μm.). (G) Quantification of plaques positive area. (H) Quantification of number of microglia. (I) mRNA level of BDNF in the cortex (*n* = 6). Data are the means ± SEM. ^*^*p* < 0.05, ^**^*p* < 0.01; one-way ANOVA with Tukey multiple comparisons test.


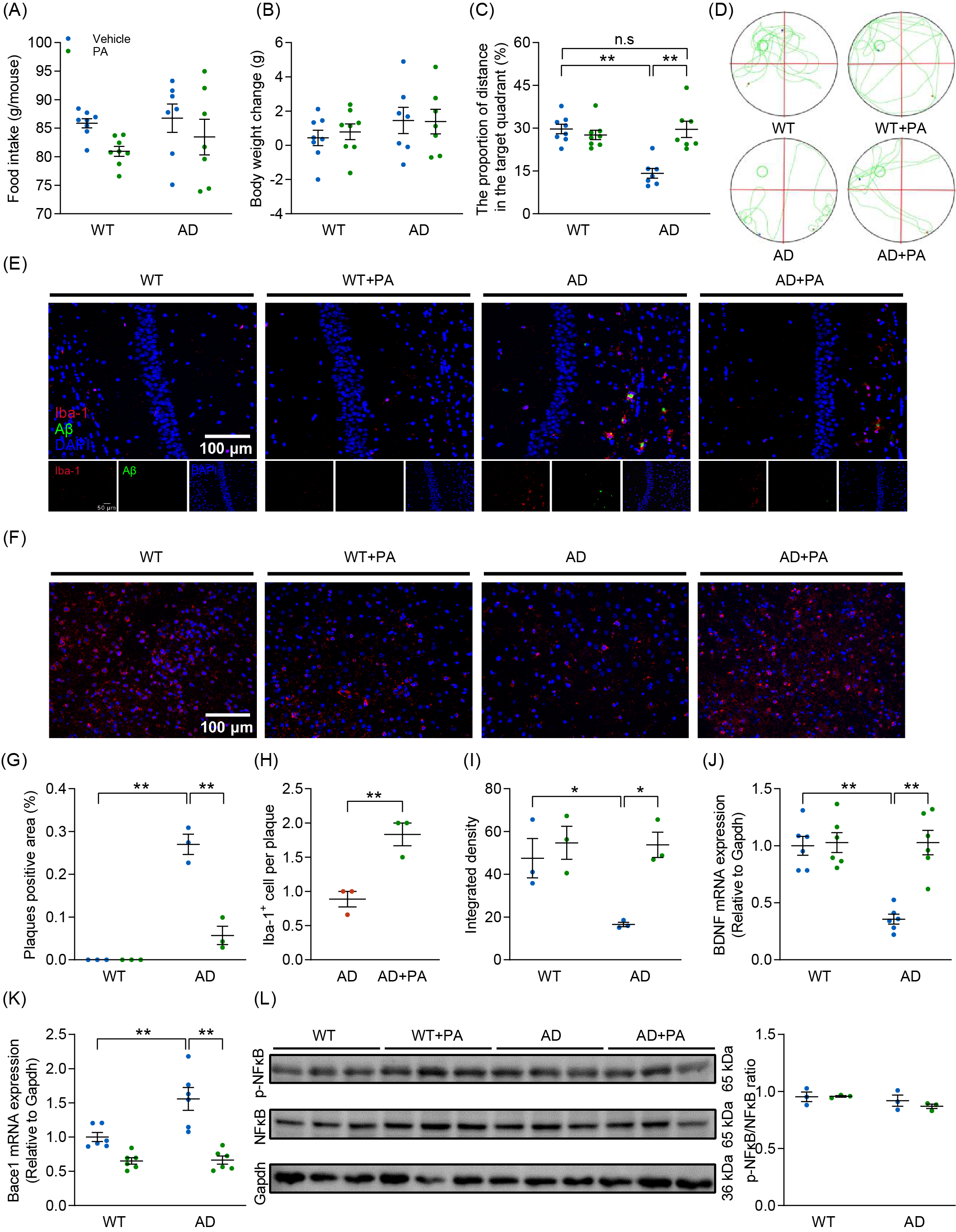
Figure S7 Propionic acid (PA) intervention mitigates AD-induced cognitive impairment. (A) Food intake (*n* = 7-8). (B) Body weight change. (C) The proportion of distance in the target quadrant. (D), Mouse trajectory during the probe trial. (E) Aβ deposition (green) and Iba-1^+^ (red) microglia immunohistochemical fluorescence images on mice hippocampal CA1 area (*n* = 3) (Scale bar, 100 μm.). (F) BDNF immunohistochemistry images on AD mice cortex (*n* = 3) (Scale bar, 100 μm.). (G) Quantification of plaques positive area. (H) Quantification of Aβ plaque-associated microglia. (I) Quantification of integrated density of BDNF. (J) mRNA level of BDNF in the cortex (*n* = 6). (K) mRNA level of β-secretase (BACE1) (*n* = 6). (L) Western blots analysis of phosphorylation of nuclear factor kappa B (p-NFκB) and NFκB (*n* = 3). Data are the means ± SEM. ^*^*p* < 0.05, ^**^*p* < 0.01; two-way ANOVA with Tukey multiple comparisons test.


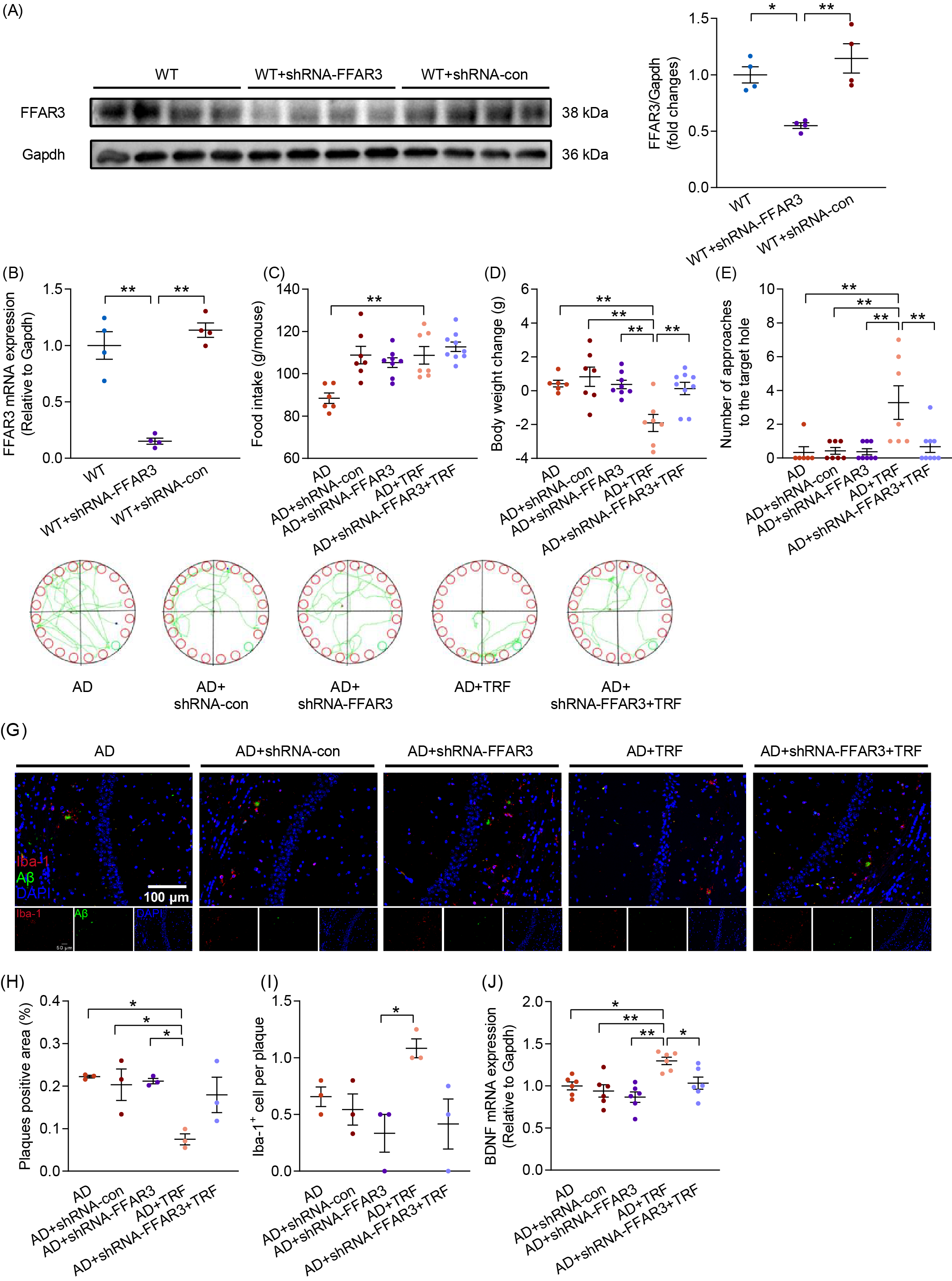
Figure S8 Knockdown of free fatty acid receptor 3 (FFAR3) eliminates the improved effect of TRF. (A) Western blots analysis of FFAR3 in mouse brain after injection of shRNA-FFAR3, shRNA-con (*n* = 4). (B) mRNA level of FFAR3. (C) Food intake (*n* = 6-9). (D) Body weight change. (E) Number of approaches to the target hole. (F), Mouse trajectory during the probe trial. (G) Aβ deposition (green) and Iba-1^+^ (red) microglia immunohistochemical fluorescence images on mice hippocampal CA1 area (*n* = 3) (Scale bar, 100 μm.). (H) Quantification of plaques positive area. (I) Quantification of Aβ plaque-associated microglia. (J) mRNA level of BDNF in the cortex (*n* = 6). Data are the means ± SEM. ^*^*p* < 0.05, ^**^*p* < 0.01; one-way ANOVA with Tukey multiple comparisons test.


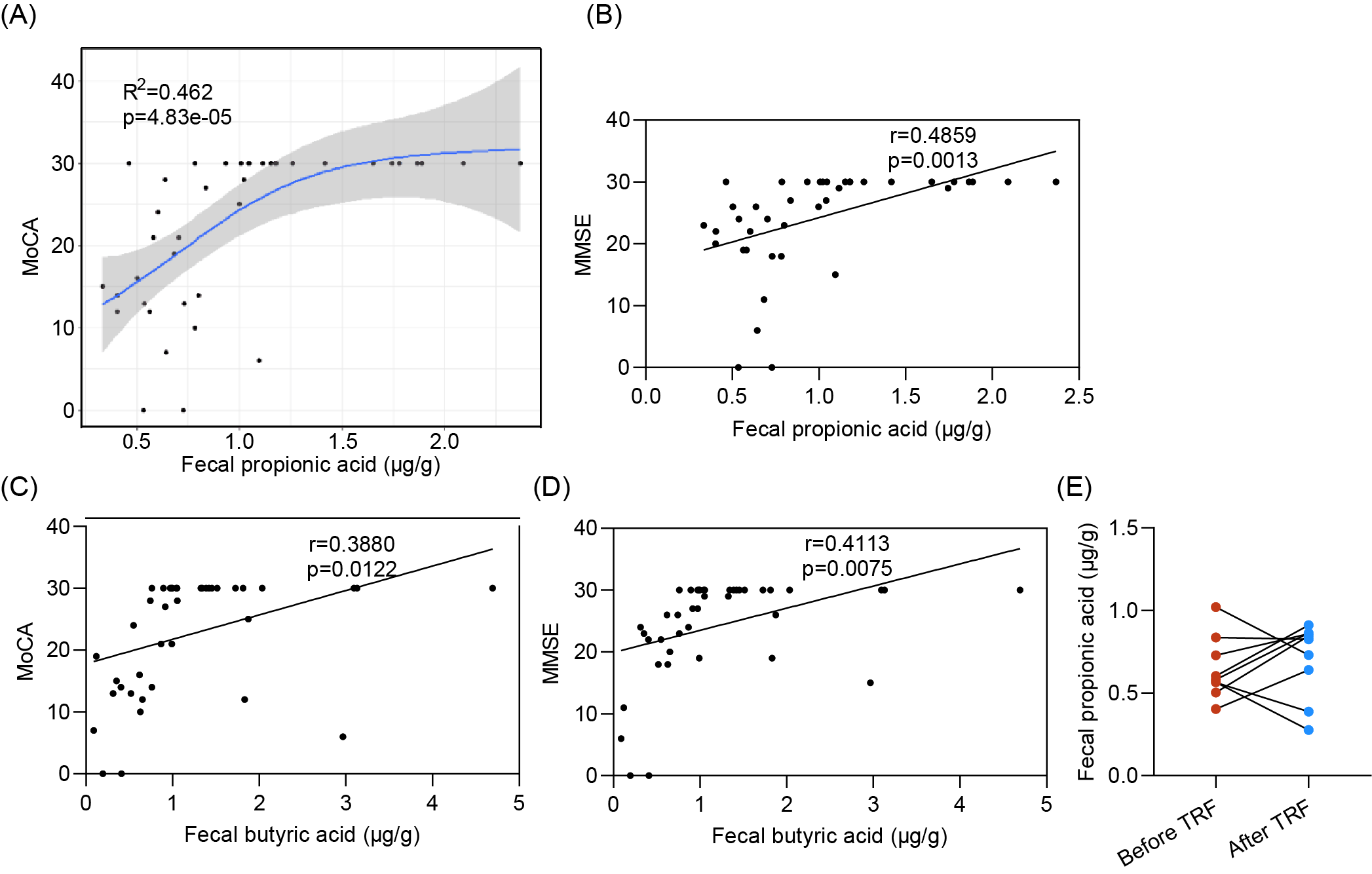
Figure S9 A case-control study of fecal SCFAs in AD patients and a clinical intervention study of TRF on AD patients. (A) Generalized additive model was constructed to assess the correlation between PA level and montreal cognitive assessment (MoCA) score using R package mgcv (*n* = 41). (B) Scatter plot showing a strong positive correlation between MMSE score and fecal PA level (Pearson correlation coefficient *r* = 0.4859, *p* = 0.0013). (C) Scatter plot showing a strong positive correlation between MoCA score and fecal butyric acid level (Pearson correlation coefficient *r* = 0.3880, *p* = 0.0122). (D) Scatter plot showing a strong positive correlation between MMSE score and fecal butyric acid level (Pearson correlation coefficient *r* = 0.4113, *p* = 0.0075). (E) Quantification of fecal PA level from AD patients before and after 4-month TRF intervention (*n* = 9). Data are assessed using two-tailed paired Student’s t-test, ^*^*p* < 0.05, ^**^*p* < 0.01.
